# Bromamine T (BAT) Exerts Stronger Anti-Cancer Properties than Taurine (Tau)

**DOI:** 10.3390/cancers13020182

**Published:** 2021-01-07

**Authors:** Stella Baliou, Maria Goulielmaki, Petros Ioannou, Christina Cheimonidi, Ioannis P. Trougakos, Markus Nagl, Anthony M. Kyriakopoulos, Vassilis Zoumpourlis

**Affiliations:** 1 Biomedical Application Unit, Institute of Chemical Biology, National Hellenic Research Foundation, 48 Vas. Constantinou Ave., 11635 Athens, Greece; smpaliou@eie.gr (S.B.); mgoulielmaki@eie.gr (M.G.); 2 Department of Internal Medicine & Infectious Diseases, University Hospital of Heraklion, 71110 Heraklion, Crete, Greece; p.ioannou@med.uoc.gr; 3 Department of Cell Biology and Biophysics, Faculty of Biology, National and Kapodistrian University of Athens, 15784 Athens, Greece; chrischeim@biol.uoa.gr (C.C.); itrougakos@biol.uoa.gr (I.P.T.); 4 Department of Hygiene, Microbiology and Public Health, Institute of Hygiene and Medical Microbiology, Medical University of Innsbruck, 6020 Innsbruck, Austria; m.nagl@i-med.ac.at; 5 Department of Research and Development, Nasco AD Biotechnology Laboratory, 11 Sachtouri Str, 18536 Piraeus, Greece; antkyriak@gmail.com

**Keywords:** bromamine T, taurine, colon cancer, breast cancer

## Abstract

**Simple Summary:**

Taurine (Tau) has been shown to inhibit cancer growth. However, the mechanisms that underlie the growth-inhibitory effects of Tau remain obscure in both colon and breast cancer. In parallel, N-bromotaurine (TauNHBr) and a stable active bromine molecule, bromamine T (BAT), appear to exert strong anti-inflammatory effects. To our knowledge, this is the first study that evaluates the anti-cancer effects of BAT and its underlying mechanisms. To gain a comprehensive picture of the cytotoxic effect of BAT on colon and breast cancer, we compared its effect with that of Tau. Our data support that BAT exerts a superior anti-cancer effect than Tau, through the induction of cell death, probably due to the activation of distinct mitogen-activated protein kinase (MAPK) family members. Interestingly, BAT inhibits colon carcinogenesis in vivo to a greater extent than Tau. Our data significantly add to the use of BAT as a novel therapeutic modality in colon and breast cancer.

**Abstract:**

Background: Taurine (Tau) ameliorates cancer pathogenesis. Researchers have focused on the functional properties of bromamine T (BAT), a stable active bromine molecule. Both N-bromotaurine (TauNHBr) and BAT exert potent anti-inflammatory properties, but the landscape remains obscure concerning the anti-cancer effect of BAT. Methods: We used Crystal Violet, colony formation, flow cytometry and Western blot experiments to evaluate the effect of BAT and Tau on the apoptosis and autophagy of cancer cells. Xenograft experiments were used to determine the in vivo cytotoxicity of either agent. Results: We demonstrated that both BAT and Tau inhibited the growth of human colon, breast, cervical and skin cancer cell lines. Among them, BAT exerted the greatest cytotoxic effect on both RKO and MDA-MB-468 cells. In particular, BAT increased the phosphorylation of c-Jun N-terminal kinases (JNK½), p38 mitogen-activated protein kinase (MAPK), and extracellular-signal-regulated kinases (ERK½), thereby inducing mitochondrial apoptosis and autophagy in RKO cells. In contrast, Tau exerted its cytotoxic effect by upregulating JNK½ forms, thus triggering mitochondrial apoptosis in RKO cells. Accordingly, colon cancer growth was impaired in vivo. Conclusions: BAT and Tau exerted their anti-tumor properties through the induction of (i) mitochondrial apoptosis, (ii) the MAPK family, and iii) autophagy, providing novel anti-cancer therapeutic modalities.

## 1. Introduction

Colorectal cancer (CRC) is one of the most prevalent carcinomas worldwide [1], with the BRAF oncogene being mutated in 5–8% of cases [2,3]. Identifying the mechanism underlying cancer cell resistance elicited by BRAF inhibitors is critical for the development of more effective therapies [4,5,6]. In this context, various chemotherapeutic agents have been proposed to exert their anti-tumor effect though irreversible DNA damage caused by reactive oxygen species (ROS) accumulation [7,8], since cancer cells are characterized by a higher metabolic rate than normal cells, rendering them more vulnerable to ROS-mediated insults and cell death pathways [9]. Excessive ROS generation appears to be harmful to cells, thereby leading them to cell death, including autophagy and apoptosis [10].

Interestingly, apoptosis is usually the major mechanism of chemotherapy-induced cell death, and pathways regulating apoptosis are under investigation [11]. Apart from apoptosis, many chemotherapeutic drugs have leveraged the autophagic process to exert their anti-tumor efficacy [12,13]. Autophagosomes are considered the hallmarks of autophagy-related cell death and are characterized by the presence of membrane-bound microtubule-associated protein 1 light chain 3 (LC3)-phospholipid conjugates [14]. Precisely, LC3-I is converted to LC3-II through lipidation by a ubiquitin-like system, involving autophagy-related genes 7 and 3 (ATG7 and ATG3), that allows LC3 to become associated with autophagic vesicles. In particular, LC3-II binds p62 which induces target proteins to be delivered to the autophagosome [15].

Taurine (chemical structure C_2_H_7_NO_3_S or NH_2_CH_2_CH_2_SO_3_H) is a non-essential amino acid that does not participate in protein synthesis, since it is devoid of a carboxyl group, while it does not constitute an energy source, since it is not metabolized or involved in gluconeogenesis [16]. The biosynthesis of taurine (Tau) occurs primarily in the liver, in the kidney, and to a smaller extent, in the brain [17,18], and relies on the metabolism of cysteine and methionine [19]. Tau is involved in osmoregulation, the modulation of protein phosphorylation, calcium ion regulation, anti-oxidant response, membrane stabilization, bile acid conjugation, lipid metabolism, glucose regulation [20]. Tau displays a strong growth-inhibitory effect on multiple cancer types including colon cancer [21,22], lung cancer [23], hepatocarcinoma [24], pancreatic cancer [25], glioma [26], melanoma [27], breast cancer [28,29,30,31], nasopharyngeal carcinoma [32], prostate cancer [33,34] and ovarian cancer [35]. Additionally, Tau has been shown to attenuate the toxic side effects of classical chemotherapeutic drugs (doxorubicin, 5-fluorouracil, cis-platin, tamoxifen), thereby enhancing their therapeutic efficacy [36,37,38,39,40]. In colon cancer, Tau has been shown to cause apoptosis to a greater extent in p53 mutant (HT-29) than p53 proficient (LoVo) colon cancer cells [21]. Tau has also been proved to induce apoptosis in p53 mutant (Caco2) colon cancer cells, not only relying on p53 but also on the MSTI-JNK signaling pathway [22]. Consistently with in vitro indications, Tau has been demonstrated to attenuate symptoms of dextran sulfate sodium (DSS)-induced experimental colitis in mice [41].

N-bromotaurine (TauNHBr) has emerged as a potential surrogate for Tau in cancer treatment. In activated neutrophils, Tau fulfills its cytoprotective properties through its reaction with hypobromous acid (HOBr), generating TauNHBr during inflammation [42]. TauNHBr has been suggested as a new therapeutic agent against glucocorticoid-resistant skin cancer cells [43]. However, TauNHBr is a relatively unstable molecule [42] and for this reason, an active bromine molecule, bromamine T (BAT) (chemical structure C_7_H_7_BrNNaO_2_S), has been designed [44]. BAT, the sodium salt of N-bromo-4-toluenesulfonamide, was prepared by the reaction of chloramine T with elemental bromine at the Institute of Hygiene and Medical Microbiology of the Medical University of Innsbruck by Waldemar Gottardi according to the method of Nair et al. [44]. Originally, it was used as an oxidimetric titrant [44]. BAT has been shown to have anti-inflammatory and antimicrobial properties that are similar to those of TauNHBr [45]. Recently, it has been shown that BAT has the typical broad-spectrum microbicidal activity of haloamines mediated by its oxidative activity and anti-inflammatory properties mediated by the downregulation of pro-inflammatory cytokines and chemokines such as interleukins and tumor necrosis factor alpha [45]. Remarkably, the toxicity of BAT is particularly low compared to that against microbes [45]. On the other hand, BAT has been used with success in a case of multi-bacterial scalp infection [46], while, it has also been used in cases of patients with acne vulgaris, showing similar effects with a commonly used antimicrobial, clindamycin [47].

The primary objective of this study was to examine whether a stable active bromine molecule (BAT) exerts anti-cancer properties like Tau and to determine the molecular mechanisms underlying the anti-cancer effect of BAT and Tau.

## 2. Results

### 2.1. The Anti-Proliferative Effect of BAT and Tau on Cancer Cell Growth

**Figure 1:**
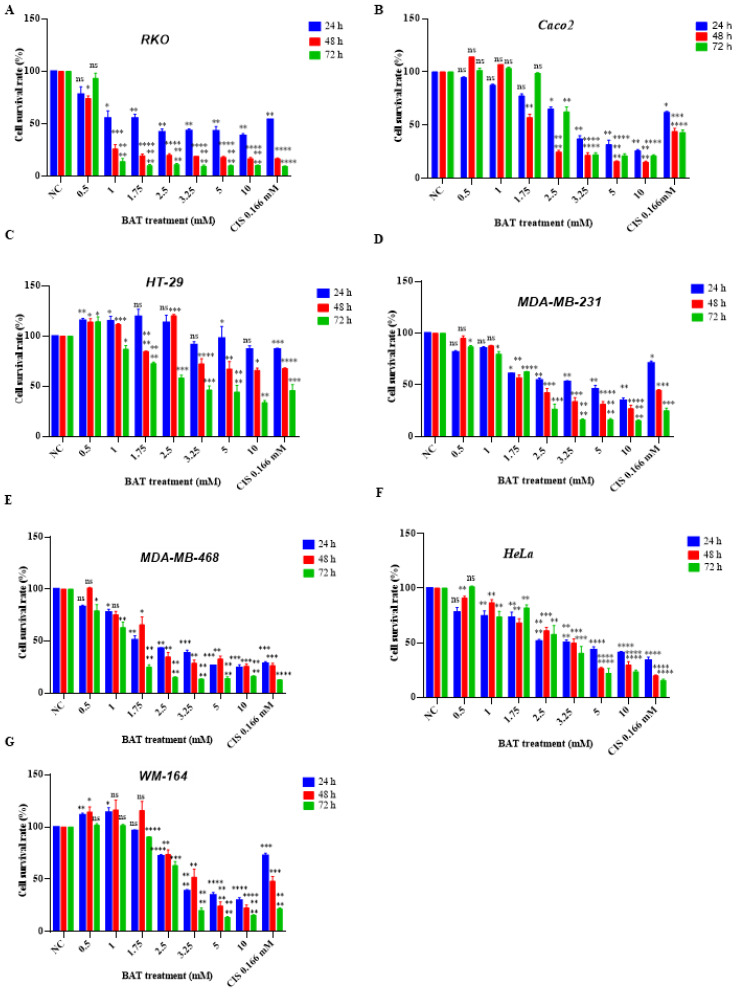
Bromamine T (BAT) is cytotoxic on a wide spectrum of cancer cells. The following cells: (**A**) RKO, (**B**) Caco2, (**C**) HT-29 (**D**) MDA-MB-231, (**E**) MDA-MB-468, (**F**) HeLa, (**G**) WM-164 cells were treated with (0.5–10 mΜ) BAT or 0.166 mM cisplatin (CIS) for 24–72 h. The percentage of viable cells was assessed upon BAT or Tau treatment versus negative control (NC) using the Crystal Violet procedure and statistical analysis was performed. ns: not significant, * *p* < 0.05. ** *p* < 0.01. *** *p* < 0.001.**** *p* < 0.0001.

**Figure 2:**
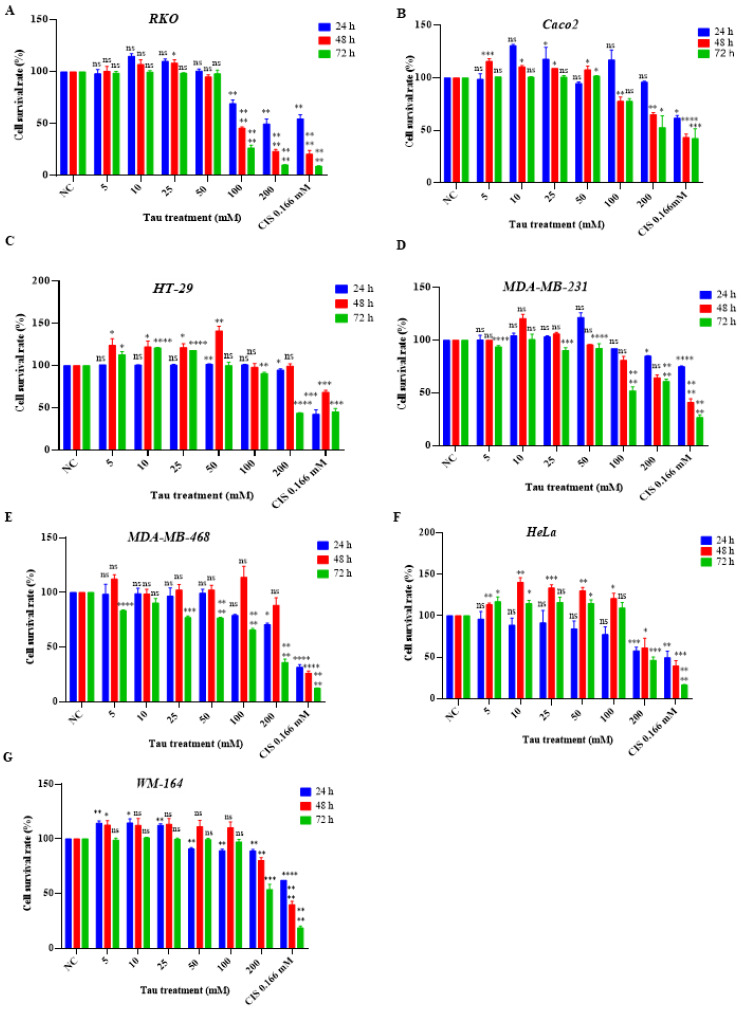
Tau is cytotoxic on a wide spectrum of cancer cells. The following cells: (**A**) RKO, (**B**) Caco2, (**C**) HT-29 (**D**) MDA-MB-231, (**E**) MDA-MB-468, (**F**) HeLa, (**G**) WM-164 cells were treated with (5–200 mΜ) Tau or 0.166 mM CIS for 24–72 h. The percentage of viable cells upon BAT or Tau treatment versus negative control (NC) was assessed, using the Crystal Violet procedure and statistical analysis was performed. ns: not significant, * *p* < 0.05. ** *p* < 0.01. *** *p* < 0.001. **** *p* < 0.0001.

**Figure 3:**
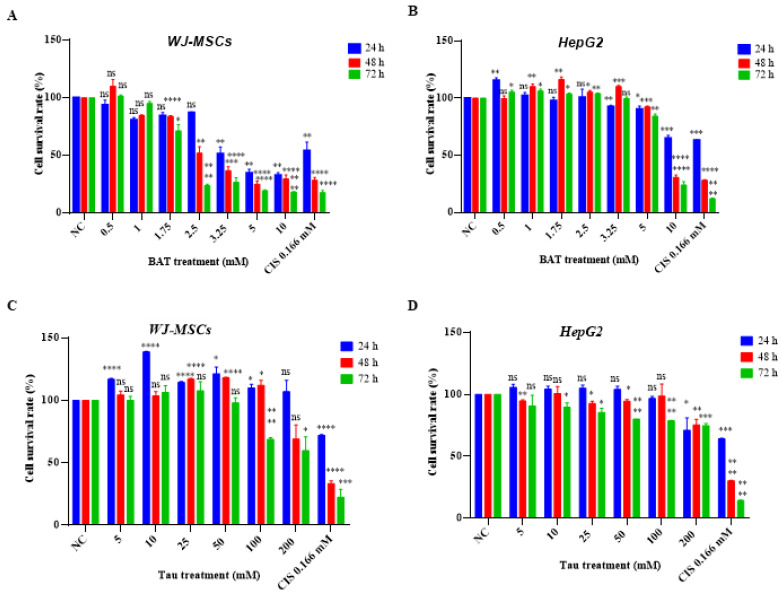
Both BAT and Tau exerted their cytotoxicity in a concentration-dependent manner. The following cells: (**A**,**C**) Wharton’s Jelly mesenchymal stem cells (WJ-MSCs) and (**B**,**D**) HepG2 cells were treated with (0.5–10 mΜ) BAT or (5–200 mΜ) Tau or 0.166 mM CIS for 24–72 h. The percentage of viable cells upon BAT or Tau treatment versus negative control (NC) was assessed, using the Crystal Violet procedure and statistical analysis was performed. ns: not significant, * *p* < 0.05. ** *p* < 0.01. *** *p* < 0.001. **** *p* < 0.0001.

To determine the cell cytotoxicity elicited by either (0.5–10 mM) BAT or (5–200 mM) Tau, human colon cancer cells (RKO, Caco2, HT-29), human breast cancer cells (MDA-MB-231, MDA-MB-468), human skin cancer cells (WM-164), and human cervical cancer cells (HeLa) were incubated with serial dilutions of BAT (Figure 1) or Tau (Figure 2) for 24–72 h and then, the Crystal Violet assay was performed. Both BAT and Tau were cytotoxic in all cancer cells in a concentration and time-dependent manner (Figure 1 and Figure 2), with the most promising results arising in RKO cells. The beneficial impact of BAT surpassed that of Tau on cancer cell proliferation in a concentration- and time-dependent manner (Tables S1–S7). For example, the viability rates of RKO cells treated with BAT at concentrations (0.5, 1, and 1.75 mM) for 48 h were the following: 74%, 26%, 20% (Figure 1) whereas the viability rates of RKO cells treated with Tau at concentrations (100, 200 mM) for 48 h were 45.6% and 23.3% (Figure 2). Interestingly, BAT at high concentrations was more cytotoxic than cisplatin (CIS), a common chemotherapeutic drug against colon cancer [48,49,50]. On the contrary, BAT treatment (0.5–1.75 mM) for 24–48 h and Tau treatment (5–100 mM) for 24–48 h did not display any cytotoxic effect on Wharton’s Jelly mesenchymal stem cells (WJ-MSCs) (Figure 3), (Table S8). Of great interest was that neither treatment with (0.5–3.25 mM) BAT for 24–72 h nor with (5–100 mM) Tau for 24–48 h exerted any sign of toxicity in HepG2 cells, thereby raising the possibility to use either agent as a putative therapeutic intervention without hepatic side effects (Figure 3), (Table S9). Our results regarding BAT and Tau in HepG2 cells were interesting since human hepatocellular carcinoma cells are used to evaluate in vitro cytotoxicity [51].

**Figure 4:**
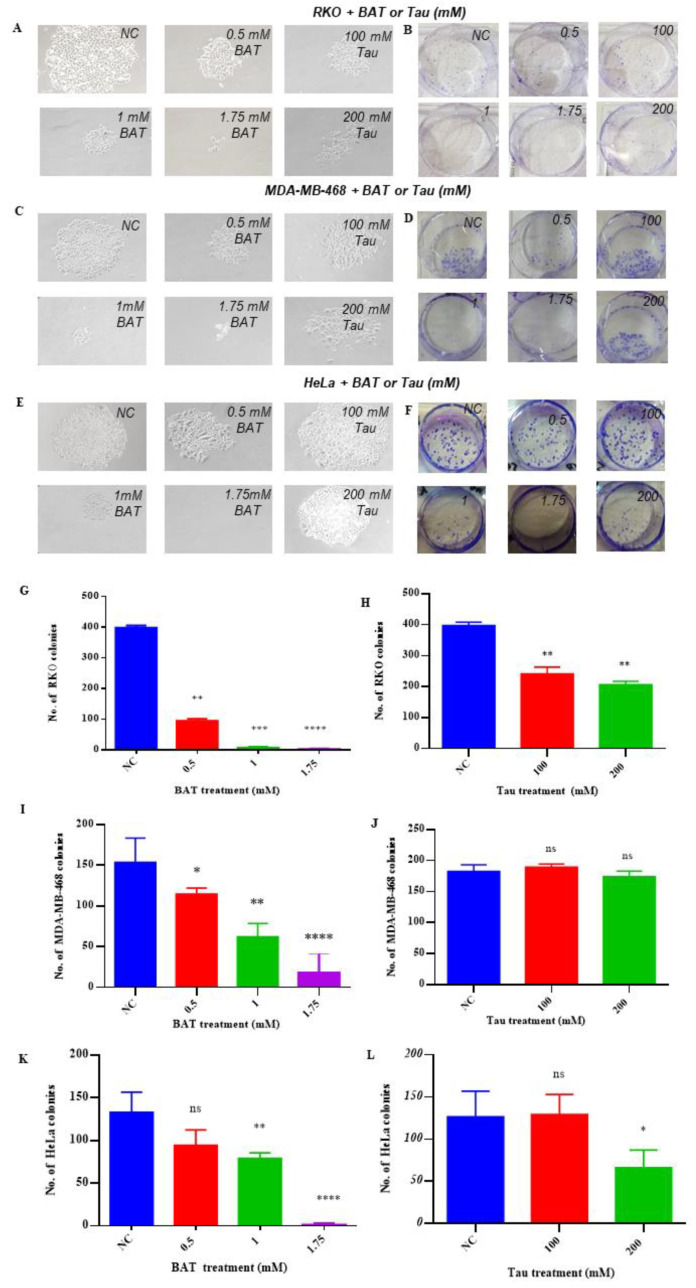
Both BAT and Tau seem to have a growth-inhibitory effect on the colon, breast, and cervical cancer cell growth in an anchorage-independent manner. Clonogenic growth images of (**A**,**B**) RKO, (**C**,**D**) MDA-MB-468, and (**E**,**F**) HeLa cells treated with (0.5–1.75 mΜ) BAT or (100–200 mM) Tau were taken after 9 days (magnification ×100). The number of colonies that occupied the area of the plate was measured, using the Promega Cell counter software. Graphs (**G**,**I**,**K**) and (**H**,**J**,**L**) represent the quantitative and statistical analysis of colony formation assays, following BAT and Tau treatment versus the negative control (NC), respectively. ns: not significant, * *p* < 0.05. ** *p* < 0.01. *** *p* < 0.001. **** *p* < 0.0001.

Based on previous results (Figure 1 and Figure 2), RKO, MDA-MB-468 cells, and HeLa were proved to be more susceptible to the cytotoxic effect of BAT or Tau treatment than other cancer cells (Caco2, HT-29, MDA-MB-231, WM-164). Our experiments further supported that both BAT and Tau hindered colon, breast, and cervical cancer cell growth in an anchorage-independent manner using the colony formation assay (Figure 4). As a result, BAT and Tau displayed a strong growth-inhibitory effect on cancer cells in both short term and long-term assays.

### 2.2. The Tumor-Inhibitory Effect of BAT and Tau through Induction of Mitochondrial Apoptosis

**Figure 5:**
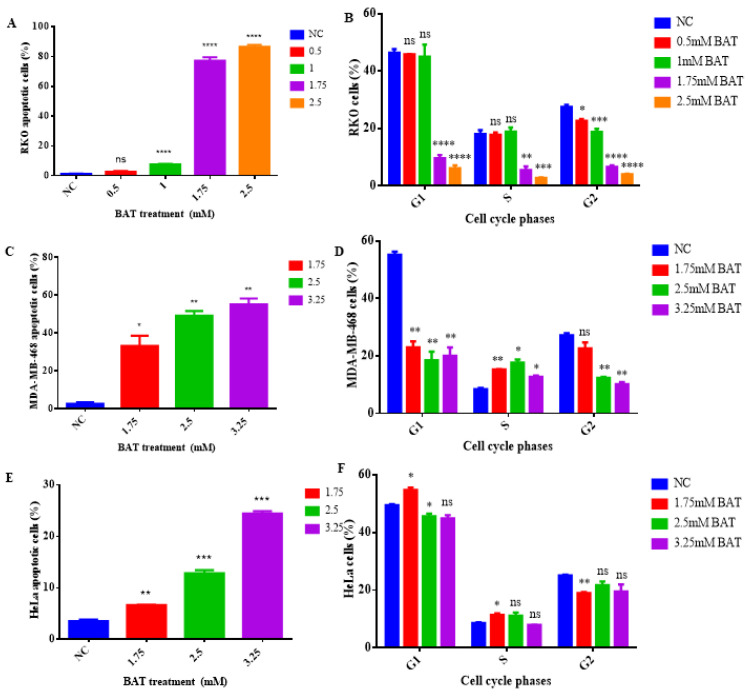

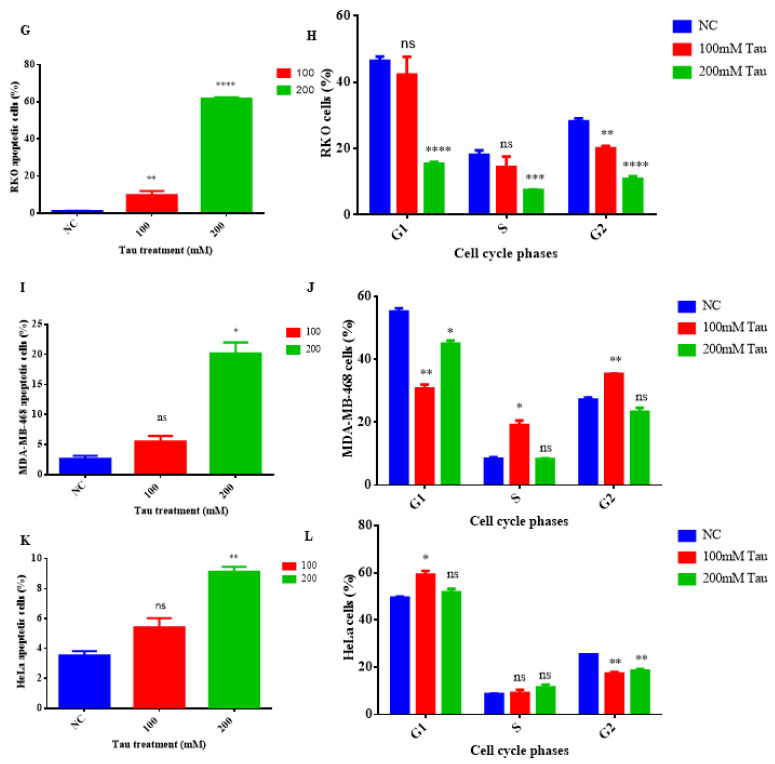
BAT and Tau do not affect cancer cell cycle regulation: RKO (**A**,**B**,**G**,**H**), MDA-MB-468 (**C**,**D**,**I**,**J**), and HeLa cells (**E**,**F**,**K**,**L**) were treated with the indicated concentrations of BAT (**A**–**F**) or Tau (**G**–**L**) for 48 h in a cell-type-dependent manner. Cells were stained with propidium iodide (PI) and they were subjected to flow cytometry (FACS) analysis to determine the cell distributions in each phase of the cell cycle in the treated groups versus negative control (NC), using BD FACS Calibur and CellQuest Pro software. Graphs (**A**,**C**,**E**) and (**G**,**I**,**K)** show the percentage of apoptotic cells and statistical analysis compared to NC, following the treatment with BAT and Tau, respectively. Graphs (**B**,**D**,**F)** and (**H**,**J**,**L**) show the percentage of cells in each phase of cell cycle of non-apoptotic cells and statistical analysis compared to NC, upon the treatment with BAT and Tau, respectively. ns: not significant, * *p* < 0.05. ** *p* < 0.01. *** *p* < 0.001, **** *p* < 0.0001.

To evaluate the apoptotic rates of BAT and Tau for 48 h, a flow cytometry (FACS) assay with the use of propidium iodide (PI) staining was conducted. Apoptotic cells were counted by FACS, excluding necrotic ones. In the FACS assay, we continued with different concentrations of either agent for 48 h in a cell-type-dependent manner (Figure 5), since the growth inhibition varied across cancer cells in a concentration-dependent manner (Figure 1 and Figure 2). In particular, BAT was used in a concentration range of (0.5–2.5 mM) in RKO cells, and in a concentration range of (1.75–3.25 mM) in MDA-MB-468 and HeLa cells (Figure 5). Our data demonstrated that BAT triggered apoptosis to a greater extent than Tau, by increasing the sub-G1 fraction of cancer cells in a cell-type-dependent manner, without eliciting any alteration in their cell cycle (Figure 5). Interestingly, the greatest percentage of apoptosis (87.3%) was observed after the treatment of RKO cells with 2.5 mM BAT for 48 h (Figure 5). Notably, the RKO cells were not treated with BAT concentrations over 2.5 mM, since maximum values were already achieved with this concentration (Figure 1) and they were also treated with BAT concentrations below 1.75 mM to identify in which phase of the cell cycle they were arrested. In contrast, RKO cells treated with 200 mM Tau exerted the greatest apoptotic effect (61.79%). The validity of FACS results was confirmed by the use of positive controls (Figure S1). Our FACS results were consistent with the apoptotic pattern and the morphological changes which were identified with the confocal microscope. It was proven that RKO cells were located at the later stages of apoptosis because they were stained positive for both annexin V-FITC and PI, in response to BAT or Tau (Figure S2). Typical features of apoptosis such as apoptotic bodies were observed.

**Figure 6:**
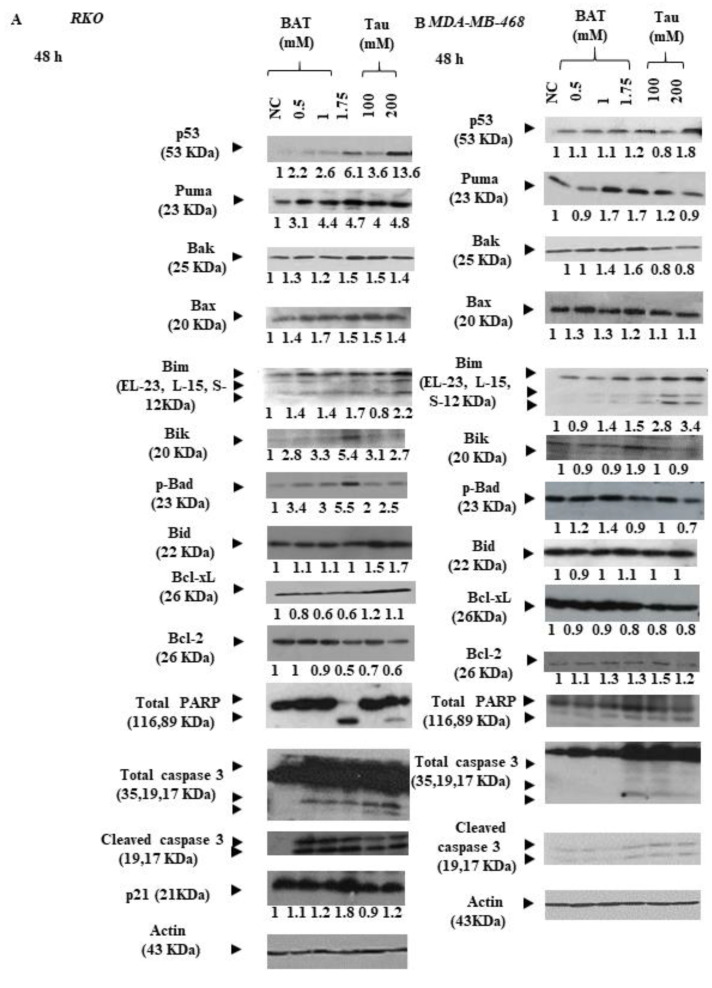

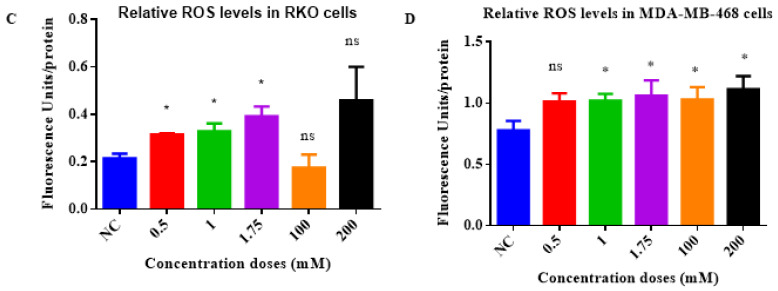
Both BAT and Tau induce mitochondrial apoptotic cell death in cancer cells. (**A**) RKO, (**B**) MDA-MB-468 cells were treated with (0.5–1.75 mM) BAT or (100–200 mM) Tau for 48 h, versus the negative control (NC). The total protein expression levels of p53, Puma, Bak, Bax, Bim, Bik, p-Bad (Ser112), Bid, Bcl-xL, Bcl-2, total PARP, total caspase 3, cleaved caspase 3 and p21 were determined by immunoblotting. β-actin was used as a loading control. The relative intensity of each molecule was compared to that of NC. The whole gel figures are shown in Figure S3 while quantification and statistical analysis are shown in Figure S5 and Table S10. (**C**,**D**) Staining with CM-H_2_DCFDA dye demonstrated the oxidative burst in RKO or MDA-MB-468 cells after treatment with (0.5–1.75 mM) BAT or (100–200 mM) Tau for 48 h, versus the negative control (NC). ns: not significant, * *p* < 0.05.

**Figure 7:**
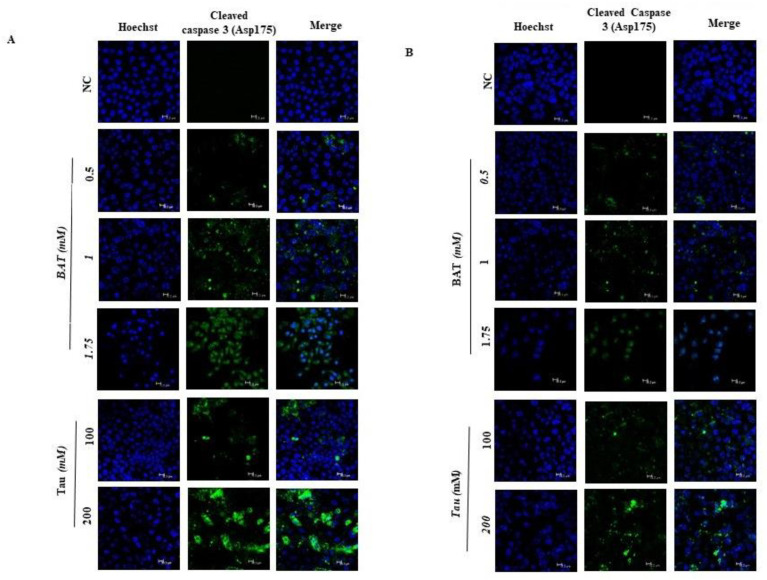
Both BAT and Tau stimulate the mitochondrial apoptotic pathway in cancer cells: (**A**) immunofluorescence staining for cleaved caspase 3 (Asp175) in RKO cells after treatment with (0.5–1.75 mM) BAT or (100–200 mM) Tau for 48 h versus negative control (NC). Hoechst was used to stain the cell nuclei (magnification ×400); (**B**) immunofluorescence staining for cleaved caspase 3 (Asp175) in MDA-MB-468 cells after treatment with (0.5–1.75 mM) BAT or (100–200 mM) Tau for 48 h versus negative control (NC). Hoechst was used to stain the cell nuclei (magnification ×400). Quantification and statistical analysis are shown in Figure S6.

Then, we aimed to delineate the molecular mechanisms underlying cell death in both RKO and MDA-MB-468 cells, following the treatment with BAT or Tau, since the aforementioned cancer cells presented the greatest growth inhibition among all cancer cells. For that reason, we focused on determining the total protein expression levels of the intrinsic mitochondrial pathway-related proteins and the extrinsic death receptor pathway-associated proteins through Western blot analysis in RKO cells (colon cancer cells with BRAF V600E mutation, p53 wild type) and in MDA-MB-468 cells (breast cancer cells with homozygous p53 mutation R273H) that had been pretreated with (0.5–1.75 mM) BAT or (100–200 mM) Tau for 48 h. In response to BAT or Tau treatment, several mitochondrial pro-apoptotic proteins like p53, PUMA (p53-upregulated modulator of apoptosis), p21 (p53 transcriptional target), Bak, Bax, and Bim were upregulated whereas Bid (extrinsic apoptotic pathway) remained stable upon BAT treatment (Figure 6A,B and Figure S5, Table S10). Interestingly, the protein expression levels of Bcl-xL and Bcl-2 (anti-apoptotic proteins) were downregulated upon BAT or Tau treatment. Importantly, the ratio of the two cleaved caspase 3 forms to full-length caspase 3 and the ratio of cleaved poly (ADP-ribose) polymerase (PARP) to full-length PARP were dramatically increased in the BAT-treated or Tau-treated RKO and MDA-MB-468 cells compared to the negative control (NC) cells. Taken together, BAT and Tau exerted their growth inhibitory action, through the activation of the mitochondrial apoptotic pathway in a cell-type-dependent manner. To evaluate the effect of BAT and Tau on apoptosis, we monitored and quantified the subcellular localization of cleaved caspase 3 (Asp175) in both RKO and MDA-MB-468 cells (Figure 7A,B and Figure S6). Our results proved that cleaved caspase-3 was markedly translocated in the nuclei in response to the RKO and MDA-MB-468 cells to BAT or Tau treatment, implying the activation of caspase-3 (Figure 7A,B and Figure S6). As a result, BAT and Tau were effective in inducing mitochondrial apoptosis in cancer cells.

### 2.3. The Effect of BAT and Tau via Elevation of ROS in Cancer Cells

Considering the well-established role of mitochondria in energy metabolism and cell death, we examined the oxidative status of cancer cells, following treatment with (0.5–1.75 mM) BAT or (100–200 mM) Tau for 48 h (Figure 6C,D). Intracellular ROS levels were significantly elevated in BAT- or Tau-treated RKO and MDA-MB-468 cells compared to NC cells, but more impressively in RKO cells (Figure 6C,D). ROS generation could function as a putative upstream regulator of mitochondrial apoptosis elicited by BAT or Tau on cancer cells, given that excessive ROS formation is often a key initiator factor that leads to apoptosis [52,53].

### 2.4. The Effect of BAT and Tau on the MAPK Pathway

**Figure 8:**
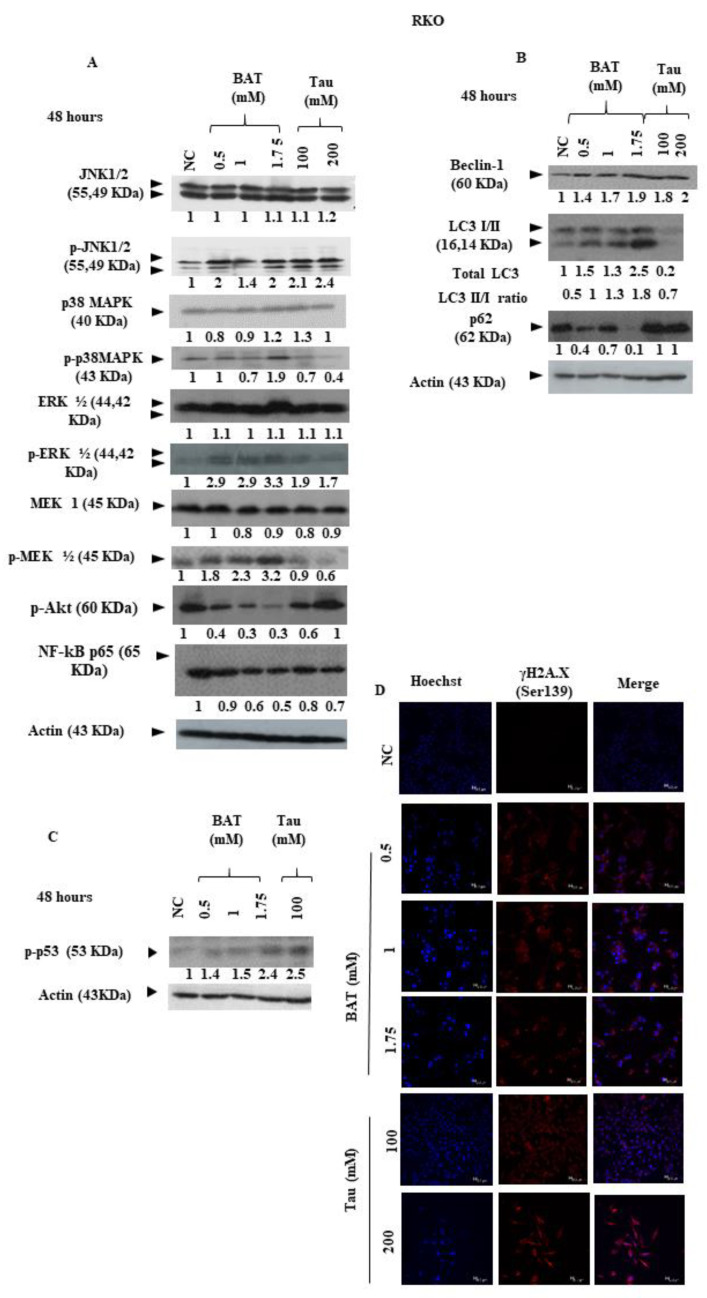
Both BAT and Tau affect the MAPK signaling pathway, autophagy, and DNA damage response (DDR) of RKO cells: (**A**) cells were treated with 0.5–1.75 mM BAT or 100–200 mM Tau for 48 h, versus negative control (NC). The total expression profiles of JNK½, p-JNK½ (Thr183/Tyr185), p38 MAPK, p-p38MAPK (Thr180/Tyr182), ERK½, p-ERK½ (Thr202/Tyr204), MEK-1, p-MEK½ (Ser217/Ser221), p-Akt (Ser473), NF-kB were determined by immunoblotting. β-actin was used as a loading control. The whole gel figures are shown in Figure S4 while quantification and statistical analysis are shown in Figure S7 and Table S10; (**B**) RKO cells were treated with (0.5–1.75 mM) BAT or (100–200 mM) Tau for 48 h, versus negative control (NC). The expression profiles of Beclin-1, LC3I/II, p62 were determined by immunoblotting. The whole gel figures are shown in Figure S4 while quantification and statistical analysis are shown in Figure S7 and Table S10. (**C**) RKO cells were treated with (0.5–1.75 mM) BAT or 100 mM Tau for 48 h, versus the negative control (NC). The expression profiles of p-p53 (Ser15) were determined by immunoblotting. β-actin was used as a loading control. The whole gel figures are shown in Figure S4 while quantification and statistical analysis are shown in Figure S7 and Table S10. (**D**) Immunofluorescence staining for γΗ2Α.X (Ser 139) after treatment with (0.5–1.75 mM) BAT or (100–200 mM) Tau for 48 h versus negative control (NC). Hoechst was used to stain the cell nuclei (magnification ×400). Quantification and statistical analysis are shown in Figure S7 and Table S10.

The MAPK pathway constitutes a well-established signal transduction pathway that determines the fate of cells, through delivering signals from upstream effectors to downstream nuclear transcription factors. To address that challenge, Western blot experiments evaluated the effect of BAT or Tau on total protein expression levels of phosphorylated p38 mitogen-activated protein kinase (MAPK) (Thr180/Tyr182), phosphorylated extracellular signal-regulated kinases (ERK½) (Thr202/Tyr 204), phosphorylated c-Jun N-terminal kinases (JNK½) (Thr183/Tyr185), phosphorylated protein kinase B (Akt) (Ser 473) and phosphorylated mitogen-activated protein (MAP)/ERK kinases (MEK½) (Ser217/Ser221) in RKO cells. In particular, the total protein expression levels of phosphorylated JNK½ and phosphorylated p38 MAPK were elevated in BAT-treated RKO cells in a concentration-dependent manner as opposed to NC cells, whereas the total protein expression levels of JNK½ and p38MAPK did not significantly change in BAT-treated RKO cells (Figure 8A and Figure S7, Table S10). In response to BAT or Tau treatment, total ERK½ protein levels remained constant (Figure 8A and Figure S7, Table S10). Notably, the total protein expression levels of phosphorylated MEK½ (Ser 217/Ser 221) were elevated in BAT-treated RKO cells compared to NC cells. In addition, the MEK½ phosphorylation was in concordance with ERK½ phosphorylation in BAT-treated RKO cells. On the other hand, phosphorylated Akt (Ser 473) was downregulated in BAT-treated RKO cells compared to NC cells. In Tau-treated RKO cells, the total protein expression levels of phosphorylated JNK½ were increased and the total protein expression levels of phosphorylated MEK½ (Ser217/Ser221) were reduced compared to NC cells. NF-kB (p65) protein expression was reduced in BAT- and Tau-treated RKO cells compared to the NC cells. As a result, the BAT and Tau seemed to mediate cell mitochondrial apoptosis under stress conditions, probably via eliciting distinct MAPK signaling pathways (Figure 8A and Figure S7, Table S10).

### 2.5. The Tumor-Suppressive Effect of BAT through Induction of Autophagy

We sought to explore any potential links between BAT or Tau action and autophagy. The expression levels of several autophagic proteins were examined in RKO cells, following treatment with (0.5–1.75 mM) BAT or (100 mM–200 mM) Tau. As depicted in Figure 8B, the ratio between the LC3II and LC3I protein levels was higher in BAT-treated RKO cells as compared to the NC cells, accompanied by the increased expression of Beclin-1, which is essential in the initial nucleation process of autophagosome formation [54] (Figure 8Β and Figure S7, Table S10). The LC3 I protein form was only converted to LC3 II in (0.5–1.75 mM) BAT-treated cells, and the conversion was not performed in (100–200 mM) Tau-treated cells (data not shown in 200 mM Tau). In Tau-treated RKO cells, only the upregulation of Beclin-1 was observed (Figure 8Β and Figure S7, Table S10). Based on the above, BAT was the only agent that enabled RKO cells to acquire autophagic properties whereas Tau treatment only triggered the initial phase of autophagy.

### 2.6. The Tumor-Inhibitory Effect of BAT and Tau on ROS-Related DNA Damage Response (DDR)

If the DNA of p53-proficient cells is subjected to ROS-mediated injury, the dissociation of p53-Mdm2 occurs, enabling the p53 transcription factor to be stabilized through post-translational modifications. Following the phosphorylation of p53 (Ser 15), the p53 transcription factor can be rescued from rapid degradation, ultimately leading to the transcriptional transactivation of p53 target genes [55]. In our results, the total protein expression levels of phosphorylated p53 (Ser 15) were increased in BAT or Tau-treated RKO cells, as opposed to those of NC cells (Figure 8C and Figure S7, Table S10). Then, to determine the effect of BAT and Tau on DDR during oxidative stress, we evaluated the total protein expression levels of phosphorylated H2A.X, given that phosphorylation of H2A.X (γH2A.X) only occurs in DNA damage response (DDR) [56]. Our data suggested that the protein content of γH2A.X was increased in BAT- and Tau-treated RKO cells compared to that of the NC cells, implying that DDR was activated following the treatment with BAT or Tau (Figure 8C,D and Figure S7, Table S10).

### 2.7. The Tumor-Inhibitory Effect of BAT and Tau In Vivo

**Figure 9:**
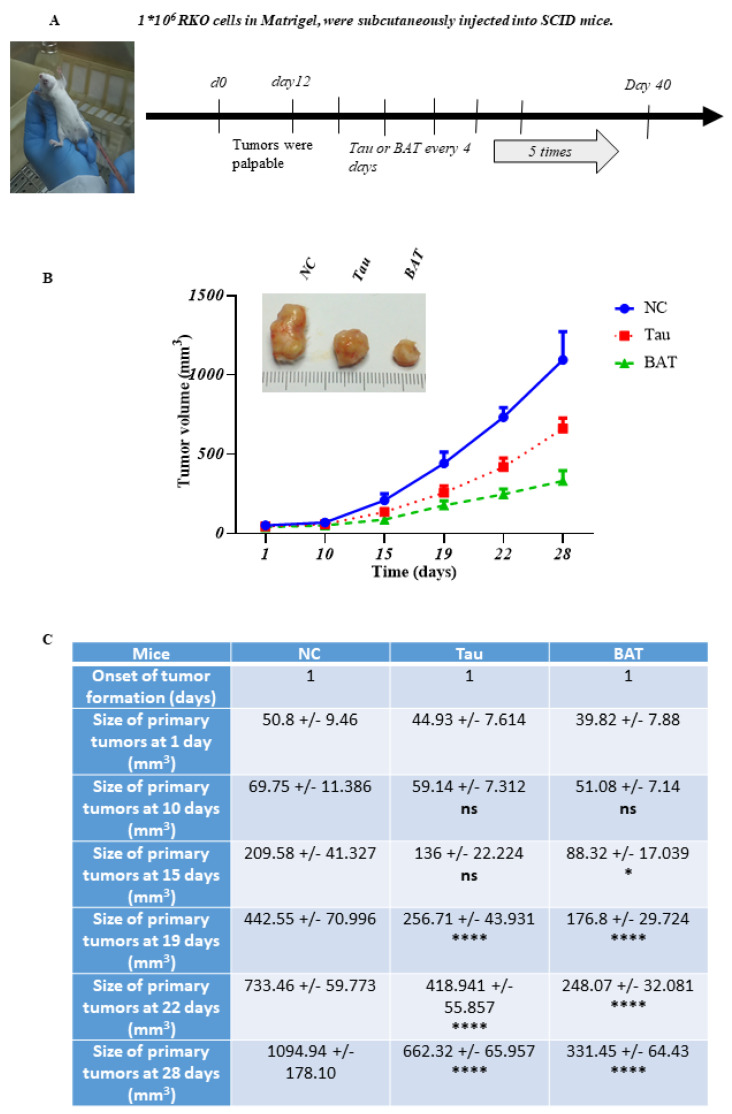
Both BAT and Tau mediate in vivo antitumor action in a xenograft model. (**A**) Schematic experimental design. A total of 1 × 10^6^ RKO cells were subcutaneously injected into the right/left flank of severe combined immune-deficient (SCID) mice (day −12). When the tumors became palpable, reaching the appropriate volume of 30–40 mm³ (day 1), the tumor-bearing mice were randomly assigned to 3 groups (6 mice/group). The first group was used as a negative control (NC) group, injected with phosphate buffer solution (PBS), and the other groups received an injection (3 mg/mouse, total 5 doses) of either agent directly into the tumor on specific days according to the timeline. Mice were sacrificed 28 days after the first day of tumor appearance (day 1). (**B**) Graph representation of the mean tumor volume in an approximately 28-day period. (**C**) Statistical analysis of the mean tumor volume between the BAT or Tau-treated groups and the negative control (NC) group. ns: not significant, * *p* < 0.05. **** *p* < 0.0001.

To shed light on the putative significance of BAT and Tau in vivo, we implanted the xenografts of RKO cells into severe combined immune-deficient (SCID) mice. When the xenograft tumors grew to the appropriate size, the mice were divided into three groups: NC, BAT-treated, and Tau-treated group. The tumor growth was monitored for 28 days after their appearance, which was considered the reference point (day 1) of the experiment (Figure 9). The BAT and Tau treatment caused an important attenuation of colon cancer progression in mice bearing RKO xenografts. Comparing the excised tumor volume of the three groups, the mean tumor volume in the BAT-treated mice was smaller compared with that of the Tau-treated mice in a statistically significant manner (Figure 9, Tables S11 and S12).

## 3. Discussion

This is, to the best of our knowledge, the first study that supports that BAT exerts a superior anti-proliferative effect in a wide spectrum of cancer cells compared to that of Tau, whereas both agents exerted minimal cytotoxicity in normal cells (WJ-MSCs) in the concentrations tested, given the high biocompatibility index of BAT [45]. Following treatment with BAT, cancer cells are prone to mitochondrial apoptosis and autophagy due to the stimulation of distinct members of the mitogen-activated protein kinase (MAPK) family. Following the treatment with Tau, cancer cells are vulnerable to mitochondrial apoptosis due to the activation of JNK½ kinases. Consistently with in vitro results, BAT exerts a stronger anti-cancer effect in vivo compared to Tau. In this way, BAT appears to be a potentially promising therapeutic agent in attenuating cancer progression, given the low tolerability of conventional therapies due to their cytotoxicity in healthy cells [57].

**Figure 10:**
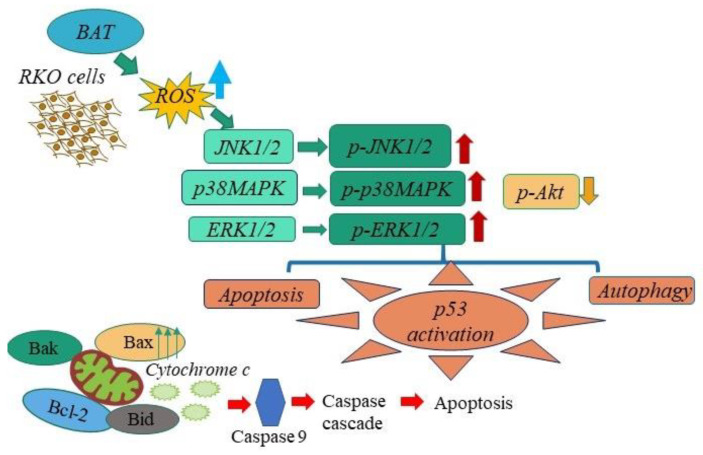
The scheme shows the underlying molecular mechanisms of BAT on colon cancer. BAT induces reactive oxygen species (ROS) accumulation, which in turn mediates mitochondrial apoptosis and autophagy. In BAT-treated RKO cells, mitochondrial apoptosis was induced through the upregulation of JNK½, p38 MAPK, ERK½ kinases, and autophagy was stimulated via the activation of JNK½ kinases as well as the downregulation of Akt.

Initially, our FACS and Western blot experiments revealed that both BAT and Tau led colon and breast cancer cells to mitochondrial apoptosis, highlighting that the effect of BAT was superior to that of Tau (Figure 5). Based on the above, the most interesting results arose in BAT- and Tau-treated RKO cells, prompting our further research to elucidate the underlying molecular mechanisms of each agent. Then, our data proved that the induction of cell death (mitochondrial and autophagic) was mediated by BAT or Tau treatment in RKO cells, via modulating the ROS-induced MAPK signaling pathways. In more detail, our data convincingly demonstrated that distinct members of the MAPK family were activated in RKO cells following the 48 h treatment with BAT or Tau. In particular, BAT caused the induction of p-JNK½, p-MEK½, p-ERK½, p-p38 MAPK, and the concomitant downregulation of p-Akt, NF-kB (p65) thereby causing both mitochondrial apoptotic and autophagic cell death in RKO cells (Figure 10). In contrast, the Tau treatment resulted in JNK½ upregulation and inhibited p-MEK½, p-ERK½, thus, mediating intrinsic apoptotic death in RKO cells. Of note, NF-kB protein levels were reduced following the treatment with BAT or Tau, confirming its inverse relationship with p21 [58] Our results are in concordance with the prevalent notion that an oxidative burst is responsible for transmitting MAPK signals, which in turn leads to the activation of cell death [59,60,61]. Hence, the cytotoxic effect of either agent was accompanied by ROS accumulation, which in turn, can induce DDR by causing DNA double-strand breaks (DSBs) [62]. Indeed, our results supported that BAT and Tau were effective in increasing the response of RKO cells to DNA damage, as evidenced by increased phosphorylated p53 (Ser15) and γH2A.X (Ser139). In our study, there was a positive correlation between the protein expression of p53 and phosphorylated p53 (Ser15), confirming a previous report that the activation of p53 through serine 15 phosphorylation can play an important role in triggering apoptosis [63]. Interestingly, our study supported that Tau might lead to ROS accumulation while, in cardiomyocytes, Tau was shown to exert its anti-oxidant properties, by contributing to the increased biosynthesis of respiratory chain complex I subunits [64], the appropriate adenosine triphosphate (ATP) generation [64], inhibition of lipid peroxidation (LPO) [65], suppression of calcium (Ca^2+^) accumulation [66], neutralization of superoxide anion (O_2_^−^) through the generation of haloamines (such as TauNHBr) [42] and the up regulation of anti-oxidant enzymes [67].

In our study, RKO cells displayed the sustained activation of the JNK½ pathway in response to BAT or Tau. Accordingly, Tau was shown to induce apoptosis, relying not only on p53 but also on the MST1-JNK signaling pathway in Caco2 cells [22]. In line with this, the JNK½ pathway has been reported to be crucial for the induction of both autophagy and apoptosis [59,68]. The underlying mechanism by which the JNK½ pathway contributes to either autophagy or apoptosis is based on the induction of phosphorylated B-cell lymphoma 2 (Bcl-2), thereby causing the dissociation of the Bcl-2/Beclin-1 [69] or Bcl-2/Bax complexes [70].

Furthermore, our data supported that BAT led to ERK½ phosphorylation, thus helping the RKO cells to induce the mitochondrial and autophagic cell death. The effect of BAT on the ERK½ signaling pathway is consistent with that of anti-tumor agents (including cisplatin [71,72,73] and taxol [74,75]) which mediates the sustained ERK½ activation to cause apoptosis [76]. In this direction, ERK½ kinases are shown to play an important role in regulating the p53 transcription factor, enabling its stabilization through the elicitation of its phosphorylation (Ser 15), promoting cell death [61]. For example, the upregulation of ERK½ kinases is crucial for p53 phosphorylation on serine 15, by preventing its assembly with Mdm2 into a complex [77]. Accordingly, it has been proven that p38 MAPK becomes activated, thus, phosphorylating several transcription factors, such as p53 [57].

Interestingly, our data provided evidence that BAT displayed anti-cancer properties through the induction of autophagy since autophagy can trigger cancer cell death apart from its established contribution in cancer cell survival [78]. In our study, JNK½ activation potentially contributed to the induction of autophagy in BAT-treated RKO cells, since ROS-mediated JNK½ activation has been reported to up-regulate autophagic proteins (Atg5, Atg7) [79,80]. 

At odds with inducing the MAPK pathway mediated by BAT treatment, BAT caused the pronounced decline of phosphorylated Akt protein expression levels. The phosphoinositide 3-kinase-Akt (PI3K/Akt) pathway usually represses both autophagy and apoptosis [81,82,83].

## 4. Materials and Methods

Taurine (chemical structure C_2_H_7_NO_3_S or NH_2_CH_2_CH_2_SO_3_H) was purchased from AppliChem ITW Companies (Taurine BioChemica, A1140,1000, LOT 3M004589). Purity was >99%, molecular weight was 125.15 g/mol. BAT (chemical structure C_7_H_7_BrNNaO_2_S) was kindly provided by Dr. Gottardi’s and Dr. Nagl’s lab (Department of Hygiene, Microbiology and Public Health, Institute of Hygiene and Medical Microbiology, Medical University of Innsbruck) (Bromamine T, Lot no. 29/06/2016). Bromamine T (N-bromo-4-toluenesulfonamide sodium, BAT × 2H_2_O) was prepared by the reaction of chloramine T with elemental bromine according to the method of Nair et al. [44]. The specifications were potency of 95.8%, bromine content of 24.83%, and effective molecular weight of 322.02 g/mol.

Cell culture: Cancer cell lines RKO, Caco2, HT-29, MDA-MB-231, MDA-MB-468, WM-164, HeLa, and HepG2 cancer cells, were obtained from our lab. All the cells were cultured in Dulbecco’s modified Eagle’s medium (DMEM, Gibco 10569-010 with 4.5 g/L D-glucose, L-glutamine, and pyruvate). All culture media were supplemented with 10% fetal bovine serum (FBS, Gibco 16000-044), and antibiotics (100 IU/mL penicillin, 100 μg/mL streptomycin). The Wharton Jelly mesenchymal stem cells (WJ-MSCs) were cultured in DMEM/F12 (with 3.5 gr/L glycine, ultraglutamine, and pyruvate), supplemented with 10% FBS, antibiotics (100 IU/mL penicillin, 100 μg/mL streptomycin), 15 mM Hepes, 1% non-essential amino acids, and 2 mM fungizone. All the cells were grown in a humidified incubator with 5% CO_2_ at 37 °C.

Crystal Violet Assay: RKO, HT-29, HeLa, WJ-MSC cells were seeded at a density of 3500 cells/well. Caco2, MDA-MB-231, MDA-MB-468, WM-164, HepG2 cells were seeded at a density of 4000 cells/well. The cells were fixed with methanol for 5 min at room temperature (RT), were resuspended with a Crystal Violet solution (0.2% Crystal Violet diluted in sddH_2_O) for 10 min at RT and were incubated with a 33% acetic acid solution. Densitometric measurement was conducted at the following wavelengths, 595 and 690 nm, using the Tecan machine. Data were presented as the mean ± SEM of three independent experiments.

Colony formation assay: The cancer cells were left to grow for 9 days in the incubator, to create colonies, and then the colonies were fixed with methanol: acetic acid solution (in 1:1 ratio), and were stained with the solution (0.5% Crystal Violet diluted in methanol). For each cell line, colony counts were corrected for plating efficiency. Images of the colonies were taken using a Nikon Eclipse T-200 inverted phase-contrast microscope equipped with an Olympus digital camera and representative images were shown in the figure. Data were presented as the mean ± SEM of three independent experiments.

Apoptosis detection by staining with propidium iodide (PI) in flow cytometry (FACS): The cells were harvested, using a 0.05% trypsin solution (Gibco, 15400-054) and by keeping the old culture medium. Then, 1.5 × 10^6^ cells were centrifuged at 1800 rpm for 5 min at RT and were suspended in 1 mL ice-cold 1× phosphate buffer solution (PBS), to break the cell clumps into single cells. The cells were fixed with ice-cold 100% ethanol at 4 °C for 4 h. After centrifugation for 5 min at RT, the cells were resuspended with the desired volume of the staining solution (50 μg/mL PI, 10 mM Tris-HCl (pH = 7.5), 5 mM MgCl_2,_ 10 μg/mL RNAse A) for 30 min at RT, in a dark room. Single cells (10,000 cells per sample) were analyzed using a FACScan flow cytometer (Becton Dickinson, San Jose, CA, USA). Data were presented as the mean ± SEM of three independent experiments.

Apoptosis detection by staining with annexin V-FITC and PI: The Annexin V-FITC Apoptosis Detection kit I (Clontech Laboratories, Inc., Mountain View, CA, USA, Protocol No. PT3050-1 2 Version No. PR983322) was used to detect apoptosis under a confocal microscope (Leica Microsystems, Weltzlar, Germany). The LAS AF program was used to acquire images and representative images were shown in the figure.

Measurement of reactive oxygen species: after trypsinization, the cells were incubated for 30 min at 37 °C with the CM-H_2_DCFDA dye (Invitrogen, Carlsbad, CA, USA). The resulting fluorescence was monitored by spectrophotometry (excitation: 490 nm, emission: 520 nm), using the VersaFluorTM Fluorometer System.

Protein extraction/Western blot analysis: Cell protein extracts were prepared, by using in Radioimmunoprecipitation assay (RIPA)/-Sodium Dodecyl Sulfate (SDS) lysis buffer containing protease inhibitors on ice and centrifuged for 15 min at 13.000 × rpm (4 °C). The composition of RIPA/-SDS buffer was: 50 mM Tris-HCl (pH = 7.5), 0.5% SDS, 1% NP-40, 150 mM NaCl, 0.25% Na-Deoxycholate, 1 mM ethylenediaminetetraacetic acid (EDTA) (pH = 8), 10% glycerol supplemented with 1mM phenylmethylsulfonyl fluoride (PMSF), 2 μg/mL Aprotinin, 1 mM NaF. The protein contents of cell or tissue lysates were adjusted by Bradford assay (Bio-Rad, Hercules, CA, USA). All samples were subjected to SDS-PAGE and transferred to the polyvinylidene difluoride (PVDF) membrane after methanol activation. Primary antibodies were applied for 12–16 h overnight at 4 °C and secondary antibodies were applied for 1 h at RT. Primary antibodies against β-actin (C4):sc-47778 Santa Cruz (Heidelberg, Germany), p53 (D0-7): sc-47698 Santa Cruz, Bax #2772 Cell Signaling (Danvers, MA, USA), Puma #4976 Cell Signaling, Bik #4592 Cell Signaling, Bim (C34C5) #2933 Cell Signaling, Bak (D4E4) #12105 Cell Signaling, Bid #2002 Cell Signaling, Caspase-3 #9662 Cell Signaling, cleaved Caspase-3 (Asp175) #9661 Cell Signaling PARP #9542 Cell Signaling, Bcl-xl (54H6) #2764 Cell Signaling, Bcl-2 (N-19):sc-492 Santa Cruz, p21 Waf1/Cip1 (12D1) #2947 Cell Signaling, p38 MAPK (D13E1) #8690 Cell Signaling, Phospho-p38 MAP kinase (Thr180/Tyr182) #9211 Cell Signaling, p44/42 MAPK (Erk½) #9102 Cell Signaling, Phospho-p44/42 MAPK (Erk½) (Thr202/Tyr204) #9101 Cell Signaling, SAPK/JNK #9252 Cell Signaling, Phospho-SAPK/JNK (Thr183/Tyr185) #9251 Cell Signaling, Phospho-p53 (Ser15) #9284 Cell Signaling, p-MEK-½ (Ser217/Ser221): #9121 Cell Signaling, MEK-1 (C-18): sc-219 Santa Cruz, Phospho-Akt (Ser473) #9271 Cell Signaling, NF-kB p65 Antibody (C-20): sc-372 Santa Cruz, Beclin-1 (H-300):sc-11427, LC3B #2775 Cell Signaling, p62 Enzo BML-PW9860. The immunoblots were developed using an enhanced chemiluminescence reagent kit (GE Healthcare Amersham, Buckinghamshire, UK), after exposure to Kodak Super RX film. The relative fold change of protein expression was analyzed using Image Studio Lite software v.5.2 and protein levels were normalized against β-actin. The used protein markers were the following: pre-stained protein standard marker-P7712S (New England Biolabs, Ipswich, MA, USA) and SeeBlue^TM^ Plus2 pre-stained protein standard marker (Invitrogen). Experiments were independently repeated three times and representative Western blots are shown in the figure.

Immunofluorescence assay: After seeding, the cells were fixed with 4% paraformaldehyde (PFA) in 1× phosphate buffer solution (PBS) pH 7.4 for 15 min at RT and they were permeabilized with 1×PBS/0.25%/Tween 20 for 10 min at RT. Then, the cells were blocked, by using 3% bovine serum albumin (BSA)-0.25%1×PBS/Tween 20 for 1 h at RT. After blocking, the cells were incubated overnight at 4 °C with a primary antibody, diluted in 1% BSA-0.25%1×PBS/Tween 20. The used primary antibodies were the following: Phospho-Histone H2A.X (Ser139) (20E3) #9718 Cell Signaling and cleaved Caspase-3 (Asp175) #9661 Cell Signaling. After washing, the cells were incubated for 1 h at RT with secondary antibody-conjugated to a fluorochrome, diluted in 1% BSA-0.25%1×PBS/Tween 20. Then, the cells were incubated with Hoechst’s pigment No. 33342 in 1×PBS for 1 min at RT. Finally, the coverslips were mounted in Prolong Gold antifade media (Molecular Probes, Eugene, OR, USA) and the cells were observed under a confocal microscope (Leica Lasertechik). The LAS AF program was used to acquire the images. Experiments were independently repeated three times and representative images are shown in the figure. Quantification of fluorescence was performed with ImageJ software.

Establishment of xenografts in mice: 1 × 10^6^ RKO cells were subcutaneously injected into the flanks of 6-week-old severe-combined immunodeficiency mice (SCID) mice. In particular, Matrigel: DMEM was used in a 3:1 ratio for the resuspension of RKO cells, that were subcutaneously injected into mice (day −12). When tumors became palpable, reaching 30–40 mm^3^ (day 1 in the graph), the mice were randomly assigned to 3 groups (6 mice/group). The first group was the NC group, in which 1×PBS was administered directly into the tumor. The other groups received either BAT or Tau, diluted in 1×PBS (3 mg/mouse, total 5 doses) directly into the tumor, based on a previously published study [21]. The tumor growth rate was recorded on specific days by measuring the major and minor axes of the formed tumors with a digital caliper. On day 40, the animals were euthanized and indicative tumors were measured. Measurements were transformed into tumor volume using the formula: tumor volume (mm^3^) = major axis × minor axis^2^ × 0.5. Data were presented as the mean ± SEM of three independent experiments. Representative images of tumor volumes derived from BAT- or Tau-treated groups versus NC groups are shown in the figure.

Statistics: Student’s unpaired t-test was used to evaluate the statistical significance of the results of the Crystal Violet assay, the colony formation assay, the ROS assay, the quantified intensity of Western blots, and the immunofluorescence experiments between NC and treatment groups. Two-way analysis of the variance (ANOVA) followed by Bonferroni’s multiple comparison test was used to assess the significance of xenografts between NC and treatment groups. Statistics were calculated with GraphPad Prism 6.0 (GraphPad Software, Inc., San Diego, CA, USA).

Mice: Male SCID mice (20 g of weight, 6 weeks of age) were obtained from the National Hellenic Research Foundation (NHRF). All experiments with mice were performed in the authorized animal house of the National Hellenic Research Foundation (License number EL 25 BIObr 031 as a breeding facility, License number EL 25 BIOsup 032 as a supply facility and License number for EL 25 BIOexp 033 as a research installation). Experiments complied with the Protocol on the Protection and Welfare of Animals, as obliged by the rules of the National Hellenic Research Foundation the regulations of the National Bioethics Committee and article 3 of the presidential decree 160/1991 (in line with 86/609/EEC directive) regarding the protection of experimental animals.

## 5. Conclusions

Overall, BAT seems to be an emerging therapeutic option, exerting a significant cytotoxic effect on RKO cells through three following mechanisms: (1) the induction of mitochondrial apoptosis as well as autophagy, (2) the accumulation of ROS and induction of DDR, and (3) the activation of p-p38, p-JNK½, and p-ERK½. In MDA-MB-468 cells, BAT also seems to cause oxidative stress-related mitochondrial apoptosis. Notably, the potential functional significance of BAT is proven in vivo, using mice bearing RKO xenografts. Therefore, our findings provide a basis for further investigations aimed at elucidating the specific therapeutic efficacy of BAT against colon and breast cancer cells.

## Data Availability

The authors confirm that the datasets analyzed during the current study are available from the corresponding author upon reasonable request.

## Supplementary Materials

The following are available online at https://www.mdpi.com/2072-6694/13/2/182/s1, Tables S1–S7: Statistical analysis of cell viability assays in cancer cells upon BAT or Tau treatment for 24–72 h. Tables S8–S9: Statistical analysis of cell viability assays in cells upon BAT or Tau treatment for 24–72 h. Figure S1: The positive controls of FACS. Figure S2. Immunofluorescence staining of RKO cells with Annexin V/ PI staining. Figure S3: Whole gel figures of apoptotic cell death in RKO and MDA-MB-468 cancer cells. Figure S4: Whole gel figures of MAPK signaling pathway, autophagy and DDR in RKO cells. Figure S5: Quantification of the protein expression of apoptotic and antiapoptotic molecules in cancer cells upon BAT or Tau treatment for 48 h. Figure S6: Quantification of cleaved caspase 3 protein expression in cancer cells upon BAT or Tau treatment for 48 h. Figure S7: Quantification of MAPK pathway, autophagy and DNA damage response (DDR) expression in RKO cells upon BAT or Tau treatment for 48 h. Table S10: Statistical analysis of Western blot and immunofluorescence assays in cancer cells upon BAT or Tau treatment for 48 h. Table S11: Statistical analysis of the in vivo results according to time per treatment group. Table S12: Statistical analysis of the in vivo results according to treatment group per time.
