# Supplementary material for: Bromamine T (BAT) Exerts Stronger Anti-Cancer Properties than Taurine (Tau)
Source: Cancers (Basel). 2021 Jan 7;13(2):182. doi: 10.3390/cancers13020182 (PMC7825693; doi:10.3390/cancers13020182)
Supplement: Supplementary file 1 [file cancers-13-00182-s001.zip › Figure S1.docx]

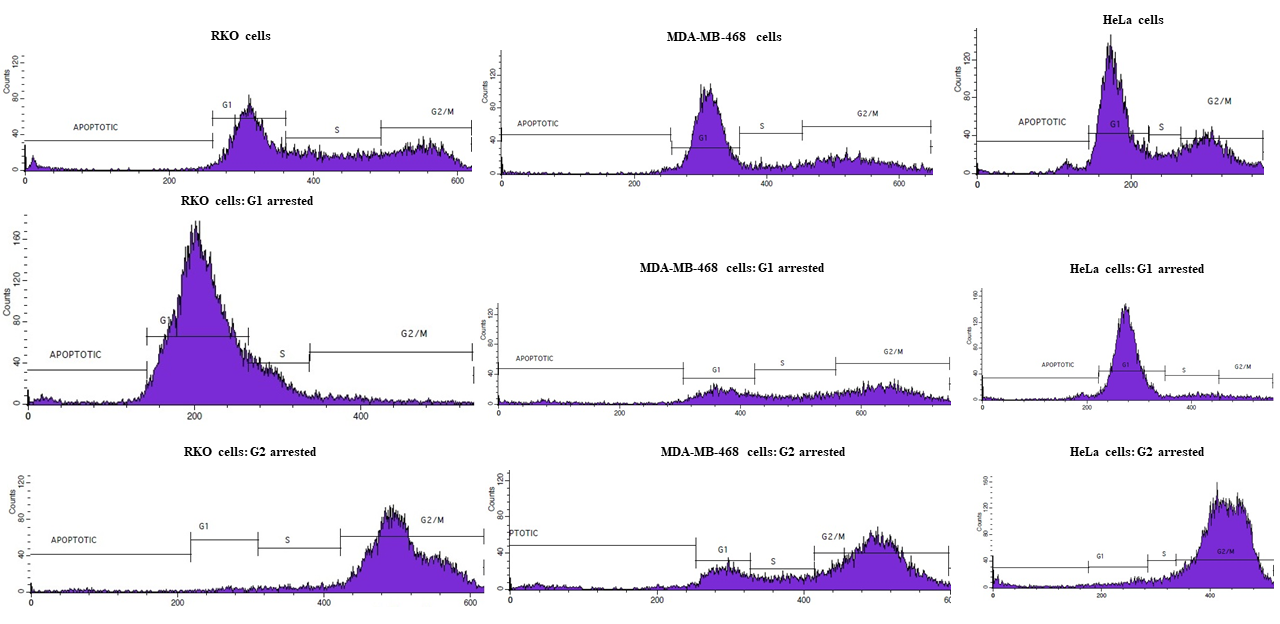


**Figure S1. The positive controls of FACS**. The reliability of FACS experiments was confirmed, by using the following substances in a specific time window: a) thymidine (2mM thymidine -18hrs, release 9hrs-2mM thymidine-17 hours) was used to disrupt the cell cycle in the G1 phase and b) nocodazole (0.4 μg/ml nocodazole-16 hours) was employed to disrupt the cell cycle in the G2 phase. Cells stained with PI were counted using BD FACS Calibur and CellQuest Pro software. Indicative images of the cell cycle of positive controls were represented.
