# Supplementary material for: Bromamine T (BAT) Exerts Stronger Anti-Cancer Properties than Taurine (Tau)
Source: Cancers (Basel). 2021 Jan 7;13(2):182. doi: 10.3390/cancers13020182 (PMC7825693; doi:10.3390/cancers13020182)
Supplement: Supplementary file 1 [file cancers-13-00182-s001.zip › Figure S2.docx]

**
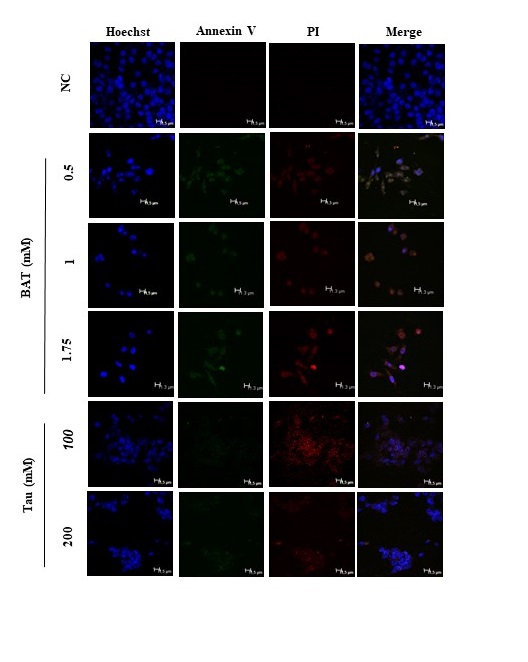
 Figure S2. Immunofluorescence staining of RKO cells with Annexin V/ PI staining**. RKO cells were treated with 0.5-1.75mΜ BAT or 100-200mM Tau for 48 hours versus negative control (NC). Microscopy images showed increased population rates at the late apoptosis stage (Annexin V ^+^ PI^+^). The apoptosis pattern was manifested by cell shrinkage and increased signals of Annexin V (green) and PI (red). Hoechst was used to stain the cell nuclei (Magnification x400).
