# Supplementary material for: Bromamine T (BAT) Exerts Stronger Anti-Cancer Properties than Taurine (Tau)
Source: Cancers (Basel). 2021 Jan 7;13(2):182. doi: 10.3390/cancers13020182 (PMC7825693; doi:10.3390/cancers13020182)
Supplement: Supplementary file 1 [file cancers-13-00182-s001.zip › Figures S5-S7.docx]

**
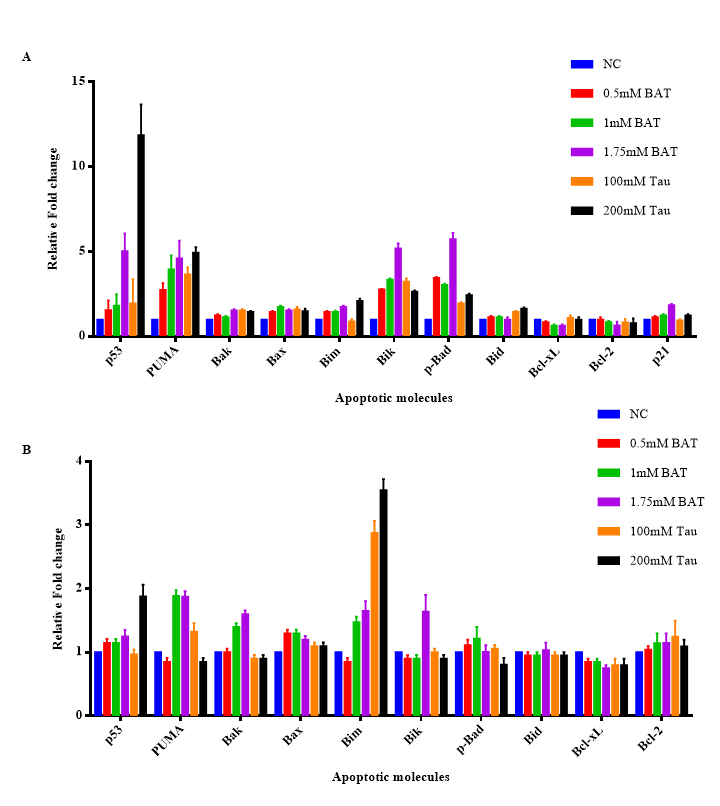
Figure S5: Quantification of protein expression of apoptotic and antiapoptotic molecules in cancer cells RKO cells upon BAT or Tau treatment for 48 hours.** The fold change in protein expression of apoptotic and antiapoptotic molecules relative to negative control (NC) is shown in RKO (A) and in MDA-MB-468 cells (B) following BAT and Tau treatment for 48 hours. Statistics show comparison of BAT and Tau treated cells with NC cells. Ns not significant, * p < 0.05. ** p < 0.01. *** p < 0.001.


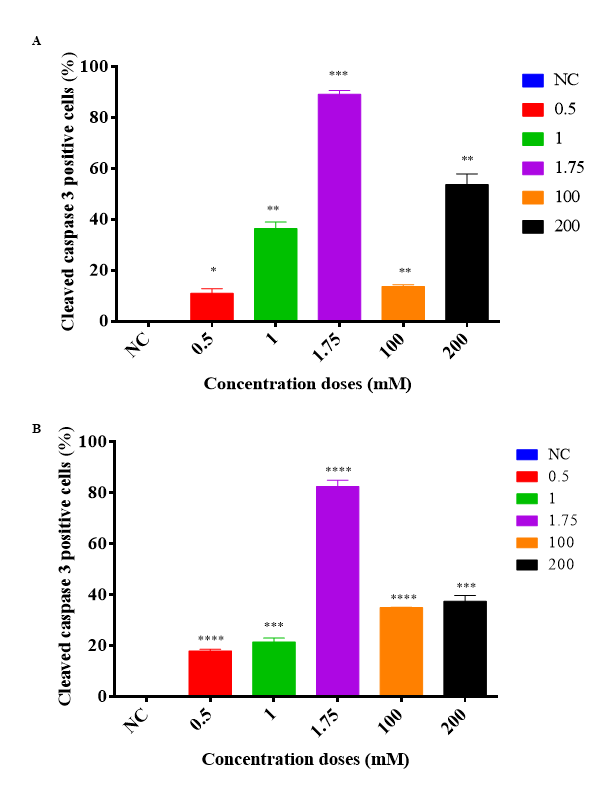


**Figure S6: Quantification of cleaved caspase 3 protein expression in cancer cells RKO cells upon BAT or Tau treatment for 48 hours.** Graph shows the percentage of cleaved caspase 3 positive cells as determined by immunofluorescence staining in RKO (A) and MDA-MB-468 cells (B) following treatment with BAT and Tau for 48 hours. Statistics show comparison of (0.5-1.75mM) BAT and (100-200mM) Tau treated cells with NC cells. Ns not significant, * p < 0.05. ** p < 0.01. *** p < 0.001, **** p<0.0001.


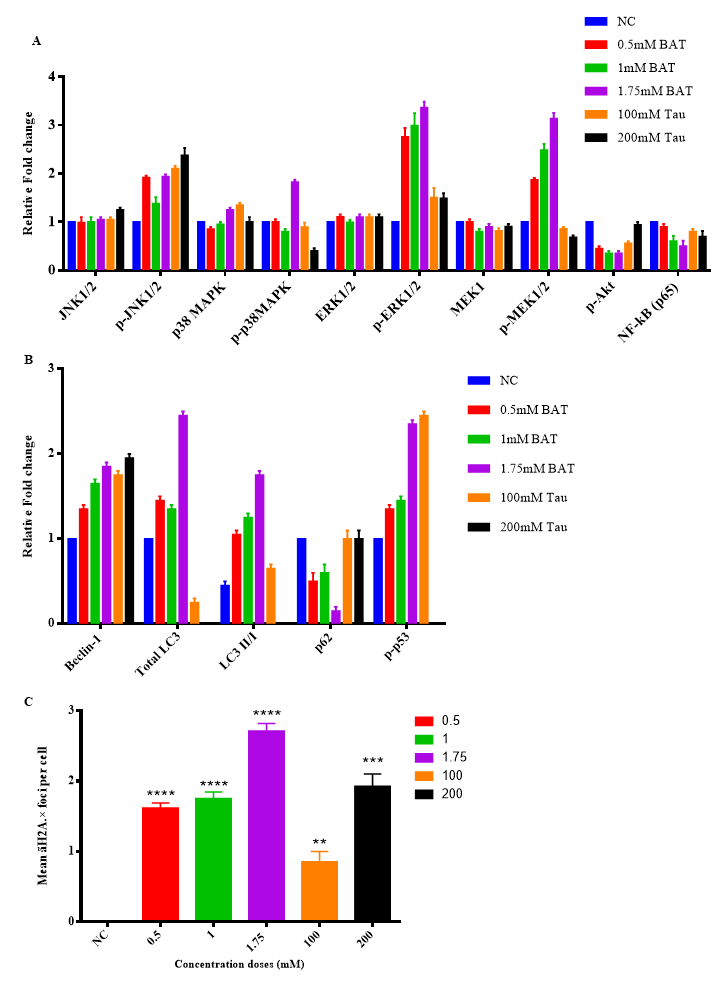


**Figure S7: Quantification of MAPK pathway, autophagy and DNA damage response (DDR) expression in RKO cells upon BAT or Tau treatment for 48 hours.** The fold change in protein expression of (A) MAPK pathway relative to negative control (NC) and (B) autophagy and DNA damage response is shown in RKO cells following BAT and Tau treatment for 48 hours. (C) Graph shows the percentage of γH2A.X foci per cell as determined by immunofluorescence staining. Statistics show comparison of (0.5-1.75mM) BAT and (100-200mM) Tau treated cells with NC cells. Ns not significant, * p < 0.05. ** p < 0.01. *** p < 0.001, **** p<0.0001.
