# Supplementary material for: Bromamine T (BAT) Exerts Stronger Anti-Cancer Properties than Taurine (Tau)
Source: Cancers (Basel). 2021 Jan 7;13(2):182. doi: 10.3390/cancers13020182 (PMC7825693; doi:10.3390/cancers13020182)
Supplement: Supplementary file 1 [file cancers-13-00182-s001.zip › Table S10.docx]

**Table S10: Statistical analysis of Western Blot and immunofluorescence assays in cancer cells upon BAT or Tau treatment for 48 hours.** Statistics show comparison of BAT and Tau treated groups and the negative control (NC). Ns not significant, *p<0.05, ** p<0.01, ***p<0.001, ****p<0.0001.

| RKO cells | 0.5mM BAT | 1mM BAT | 1.75mM BAT | 100mM Tau | 200mM Tau |
| --- | --- | --- | --- | --- | --- |
| p53 | ns | ns | ** | ns | *** |
| PUMA | ** | ** | ** | *** | **** |
| Bak | * | ns | ** | ** | * |
| Bax | * | ** | ** | * | * |
| Bim | * | * | ** | ** | ** |
| Bik | *** | *** | ** | ** | *** |
| p-Bad | *** | *** | ** | ** | ** |
| Bid | ns | ns | ns | * | ** |
| Bcl-xL | ns | * | * | ns | ns |
| Bcl-2 | ns | ns | ns | ns | ns |
| p21 | ns | * | ** | ns | * |

| MDA-MB-468 cells | 0.5mM BAT | 1mM BAT | 1.75mM BAT | 100mM Tau | 200mM Tau |
| --- | --- | --- | --- | --- | --- |
| p53 | * | * | ns | ns | ns |
| PUMA | ns | ns | ns | ns | ns |
| Bak | ns | * | ** | ns | ns |
| Bax | ** | ** | * | ns | ns |
| Bim | ns | ** | ** | **** | **** |
| Bik | ns | ns | * | ns | ns |
| p-Bad | ns | ns | ns | ns | ns |
| Bid | ns | ns | ns | ns | ns |
| Bcl-xL | ns | ns | * | ns | Ns |
| Bcl-2 | ns | ns | ns | ns | ns |

| RKO cells | 0.5mM BAT | 1mM BAT | 1.75mM BAT | 100mM Tau | 200mM Tau |
| --- | --- | --- | --- | --- | --- |
| JNK1/2 | ns | ns | ns | ns | * |
| p-JNK1/2 | **** | * | **** | **** | *** |
| p38MAPK | ns | * | * | * | ns |
| p-p38MAPK | ns | * | *** | ns | *** |
| ERK1/2 | ns | ns | ns | ns | ns |
| p-ERK1/2 | *** | ** | **** | ns | * |
| MEK1/2 | ns | * | ns | * | ns |
| p-MEK1/2 | **** | *** | **** | * | ** |
| p-Akt | ** | ** | ** | ** | ns |
| NF-kB (p65) | *** | * | * | * | ns |

| RKO cells | 0.5mM BAT | 1mM BAT | 1.75mM BAT | 100mM Tau | 200mM Tau |
| --- | --- | --- | --- | --- | --- |
| Beclin-1 | * | ** | ** | ** | ** |
| Total LC3 | * | * | ** | ** |  |
| LC3 II/I | * | ** | ** | ns |  |
| p62 | * | ns | ** | ns | ns |
| p-p53 | * | * | ** | ** |  |

| RKO cells | 0.5mM BAT | 1mM BAT | 1.75mM BAT | 100mM Tau | 200mM Tau |
| --- | --- | --- | --- | --- | --- |
| Beclin-1 | * | ** | ** | ** | ** |
| p62 | * | ns | ** | ns | ns |

| RKO cells | 0.5mM BAT | 1mM BAT | 1.75mM BAT | 100mM Tau |
| --- | --- | --- | --- | --- |
| Total LC3 | * | * | ** | ** |
| LC3 II/I | * | ** | ** | ns |
| p-p53 | * | * | ** | ** |
