# Supplementary material for: Bromamine T (BAT) Exerts Stronger Anti-Cancer Properties than Taurine (Tau)
Source: Cancers (Basel). 2021 Jan 7;13(2):182. doi: 10.3390/cancers13020182 (PMC7825693; doi:10.3390/cancers13020182)
Supplement: Supplementary file 1 [file cancers-13-00182-s001.zip › Table S11.docx]

**Table S10. Statistical analysis of the *in vivo* results according to time per treatment group.** Statistical analysis was performed using two-way ANOVA analysis. Ns not significant, *p<0.05, ** p<0.01, ***p<0.001, ****p<0.0001.

|  |  |  |  | **Time** | | | |
| --- | --- | --- | --- | --- | --- | --- | --- |
| **Time** | **Treatment group** | **Day 1** | **Day 10** | **Day 15** | **Day 19** | **Day 22** | **Day 28** |
| **Day 1** | NC |  | ns | *** | ****  ****  *** | ****  ****  **** | ****  ****  **** |
|  | Tau |  | ns | ns |  |  |  |
|  | BAT |  | ns | ns |  |  |  |
| **Day 10** | NC | ns |  | ** | ****  ****  ** | ****  ****  **** | ****  ****  **** |
|  | Tau | ns |  | ns |  |  |  |
|  | BAT | ns |  | ns |  |  |  |
| **Day 15** | NC | *** | ** |  | **** | **** | **** |
|  | Tau | ns | ns |  | * | **** | **** |
|  | BAT | ns | ns |  | ns | **** | **** |
| **Day 19** | NC | **** | **** | **** |  | **** | **** |
|  | Tau | **** | **** | * |  | *** | **** |
|  | BAT | *** | ** | ns |  | ns | **** |
| **Day 22** | NC | **** | **** | **** | **** |  | **** |
|  | Tau | **** | **** | **** | *** |  | **** |
|  | BAT | **** | **** | **** | ns |  | ns |
| **Day 28** | NC | **** | **** | **** | **** | **** |  |
|  | Tau | **** | **** | **** | **** | **** |  |
|  | BAT | **** | **** | **** | **** | ns |  |

BAT: bromamine T, Tau: taurine, ns: p>0.05, * p<0.05, ** p<0.01, *** p<0.001
