# Supplementary material for: Bromamine T (BAT) Exerts Stronger Anti-Cancer Properties than Taurine (Tau)
Source: Cancers (Basel). 2021 Jan 7;13(2):182. doi: 10.3390/cancers13020182 (PMC7825693; doi:10.3390/cancers13020182)
Supplement: Supplementary file 1 [file cancers-13-00182-s001.zip › Table S12.docx]

**Table S12. Statistical analysis of the *in vivo* results according to treatment group per time.** Statistical analysis was performed using two-way ANOVA analysis. Ns not significant, *p<0.05, ** p<0.01, ***p<0.001, ****p<0.0001.

|  |  | **Treatment group** | | |
| --- | --- | --- | --- | --- |
| **Treatment group** | **Time** | **NC** | **Tau** | **BAT** |
| **NC** | Day 10 | ns | ns | ns |
|  | Day 15 | ns | ns | ns |
|  | Day 19 | ns | ns | ns |
|  | Day 22 | ns | ns | ns |
|  | Day 28 | ns | ns | ns |
| **Tau** | Day 10 | ns | ns | ns |
|  | Day 15 | ns | ns | ns |
|  | Day 19 | **** | ns | ns |
|  | Day 22 | **** | ns | **** |
|  | Day 28 | **** | ns | **** |
| **BAT** | Day 10 | ns | ns | ns |
|  | Day 15 | * | ns | ns |
|  | Day 19 | **** | ns | ns |
|  | Day 22 | **** | **** | ns |
|  | Day 28 | **** | **** | ns |

BAT: bromamine T, Tau: taurine, ns: p>0.05, * p<0.05, ** p<0.01, *** p<0.001

|  |
| --- |
|  |
