# Supplementary material for: Bromamine T (BAT) Exerts Stronger Anti-Cancer Properties than Taurine (Tau)
Source: Cancers (Basel). 2021 Jan 7;13(2):182. doi: 10.3390/cancers13020182 (PMC7825693; doi:10.3390/cancers13020182)
Supplement: Supplementary file 1 [file cancers-13-00182-s001.zip › Tables S1-S7.docx]

**Tables** **S1-S7. Statistical analysis of cell viability assays in cancer cells upon BAT or Tau treatment for 24-72 hours.** Statistics show comparison of BAT and Tau treated groups with negative control (NC). Ns not significant, *p<0.05, ** p<0.01, ***p<0.001, ****p<0.0001.

1. **RKO cells**

| Bromamine T (BAT) | 0.5mM | 1mM | 1.75mM | 2.5mM | 3.25mM | 5mM | 10mM | CIS 0.166mM |
| --- | --- | --- | --- | --- | --- | --- | --- | --- |
| 24 hours | ns | * | ** | ** | ** | ** | ** | ** |
| 48 hours | * | *** | **** | **** | **** | **** | **** | **** |
| 72 hours | ns | **** | **** | **** | **** | **** | **** | **** |

| Taurine (Tau) | 5mM | 10mM | 25mM | 50mM | 100mM | 200mM | CIS 0.166mM |
| --- | --- | --- | --- | --- | --- | --- | --- |
| 24 hours | ns | ns | ns | ns | ** | ** | ** |
| 48 hours | ns | ns | * | ns | **** | **** | **** |
| 72 hours | ns | ns | ns | ns | **** | **** | **** |

1. **Caco2 cells**

| Bromamine T (BAT) | 0.5mM | 1mM | 1.75mM | 2.5mM | 3.25mM | 5mM | 10mM | CIS 0.166mM |
| --- | --- | --- | --- | --- | --- | --- | --- | --- |
| 24 hours | ns | ns | ns | * | ** | ** | ** | * |
| 48 hours | ns | ns | ** | **** | **** | **** | **** | *** |
| 72 hours | ns | ns | ns | ** | **** | **** | **** | **** |

| Taurine (Tau) | 5mM | 10mM | 25mM | 50mM | 100mM | 200mM | CIS 0.166mM |
| --- | --- | --- | --- | --- | --- | --- | --- |
| 24 hours | ns | ns | * | ns | ns | ns | * |
| 48 hours | *** | * | * | * | ** | ** | **** |
| 72 hours | ns | ns | ns | * | ns | * | *** |

1. **ΗΤ-29 cells**

| Bromamine T (BAT) | 0.5mM | 1mM | 1.75mM | 2.5mM | 3.25mM | 5mM | 10mM | CIS 0.166mM |
| --- | --- | --- | --- | --- | --- | --- | --- | --- |
| 24 hours | ** | * | ns | ns | ns | * | ns | *** |
| 48 hours | * | *** | **** | *** | **** | ** | * | **** |
| 72 hours | * | * | **** | *** | *** | **** | ** | *** |

| Taurine (Tau) | 5mM | 10mM | 25mM | 50mM | 100mM | 200mM | CIS 0.166mM |
| --- | --- | --- | --- | --- | --- | --- | --- |
| 24 hours | ns | ns | ns | ** | ns | * | *** |
| 48 hours | * | * | * | ** | ns | ns | *** |
| 72 hours | * | **** | **** | ns | ** | **** | *** |

1. **MDA-MB-468 cells**

| Bromamine T (BAT) | 0.5mM | 1mM | 1.75mM | 2.5mM | 3.25mM | 5mM | 10mM | CIS 0.166mM |
| --- | --- | --- | --- | --- | --- | --- | --- | --- |
| 24 hours | ns | * | ** | ** | *** | *** | *** | *** |
| 48 hours | ns | ns | * | ** | ** | ** | *** | *** |
| 72 hours | * | ** | **** | **** | **** | **** | **** | **** |

| Taurine (Tau) | 5mM | 10mM | 25mM | 50mM | 100mM | 200mM | CIS 0.166mM |
| --- | --- | --- | --- | --- | --- | --- | --- |
| 24 hours | ns | ns | ns | ns | ns | * | **** |
| 48 hours | ns | ns | ns | ns | ns | ns | **** |
| 72 hours | **** | ns | *** | **** | **** | **** | **** |

1. **MDA-MB-231 cells**

| Bromamine T (BAT) | 0.5mM | 1mM | 1.75mM | 2.5mM | 3.25mM | 5mM | 10mM | CIS 0.166mM |
| --- | --- | --- | --- | --- | --- | --- | --- | --- |
| 24 hours | ns | ns | * | ** | ** | ** | ** | * |
| 48 hours | ns | ns | ** | *** | *** | **** | **** | *** |
| 72 hours | * | * | **** | *** | **** | **** | **** | *** |

| Taurine (Tau) | 5mM | 10mM | 25mM | 50mM | 100mM | 200mM | CIS 0.166mM |
| --- | --- | --- | --- | --- | --- | --- | --- |
| 24 hours | ns | ns | ns | ns | ns | * | **** |
| 48 hours | ns | ns | ns | ns | ns | ns | **** |
| 72 hours | **** | ns | *** | **** | **** | **** | **** |

1. **HeLa cells**

| Bromamine T (BAT) | 0.5mM | 1mM | 1.75mM | 2.5mM | 3.25mM | 5mM | 10mM | CIS 0.166mM |
| --- | --- | --- | --- | --- | --- | --- | --- | --- |
| 24 hours | ns | ** | ** | **** | **** | **** | **** | **** |
| 48 hours | ** | ** | ** | *** | *** | **** | **** | **** |
| 72 hours | ns | ** | ** | ** | *** | **** | **** | **** |

| Taurine (Tau) | 5mM | 10mM | 25mM | 50mM | 100mM | 200mM | CIS 0.166mM |
| --- | --- | --- | --- | --- | --- | --- | --- |
| 24 hours | ns | ns | ns | ns | ns | *** | ** |
| 48 hours | ** | ** | *** | ** | * | * | *** |
| 72 hours | * | * | ns | * | ns | *** | **** |

1. **WM-164 cells**

| Bromamine T (BAT) | 0.5mM | 1mM | 1.75mM | 2.5mM | 3.25mM | 5mM | 10mM | CIS 0.166mM |
| --- | --- | --- | --- | --- | --- | --- | --- | --- |
| 24 hours | ** | * | ns | **** | **** | **** | **** | *** |
| 48 hours | * | ns | ns | ** | ** | **** | **** | *** |
| 72 hours | ns | ns | **** | *** | **** | **** | **** | **** |

| Taurine (Tau) | 5mM | 10mM | 25mM | 50mM | 100mM | 200mM | CIS 0.166mM |
| --- | --- | --- | --- | --- | --- | --- | --- |
| 24 hours | ** | * | ** | ** | ** | ** | **** |
| 48 hours | * | ns | ns | ns | ns | ** | **** |
| 72 hours | ns | ns | ns | ns | ns | *** | **** |
