# Supplementary material for: Bromamine T (BAT) Exerts Stronger Anti-Cancer Properties than Taurine (Tau)
Source: Cancers (Basel). 2021 Jan 7;13(2):182. doi: 10.3390/cancers13020182 (PMC7825693; doi:10.3390/cancers13020182)
Supplement: Supplementary file 1 [file cancers-13-00182-s001.zip › Tables S8-S9.docx]

**Tables S8-S9. Statistical analysis of cell viability assays in cells upon BAT or Tau treatment for 24-72 hours.** Statistics show comparison of BAT and Tau treated groups with negative control (NC). Ns not significant, *p<0.05, ** p<0.01, ***p<0.001, ****p<0.0001.

1. **WJ-MSCs**

| Bromamine T (BAT) | 0.5mM | 1mM | 1.75mM | 2.5mM | 3.25mM | 5mM | 10mM | CIS 0.166mM |
| --- | --- | --- | --- | --- | --- | --- | --- | --- |
| 24 hours | ns | ns | ns | ns | ** | ** | ** | ** |
| 48 hours | ns | ns | **** | ** | *** | **** | **** | **** |
| 72 hours | ns | ns | * | **** | **** | **** | **** | **** |

| Taurine (Tau) | 5 mM | 10 mM | 25 mM | 50 mM | 100 mM | 200 mM | CIS 0.166 mM |
| --- | --- | --- | --- | --- | --- | --- | --- |
| 24 hours | **** | **** | **** | * | * | ns | **** |
| 48 hours | ns | ns | **** | **** | * | ns | **** |
| 72 hours | ns | ns | ns | ns | **** | * | *** |

**9. HepG2 cells**

| Bromamine T (BAT) | 0.5mM | 1mM | 1.75mM | 2.5mM | 3.25mM | 5mM | 10mM | CIS 0.166mM |
| --- | --- | --- | --- | --- | --- | --- | --- | --- |
| 24 hours | ** | ns | ns | ns | ** | * | *** | *** |
| 48 hours | ns | ** | ** | * | *** | *** | **** | **** |
| 72 hours | * | * | * | ** | ns | ** | **** | **** |

| Taurine (Tau) | 5mM | 10mM | 25mM | 50mM | 100mM | 200mM | CIS 0.166mM |
| --- | --- | --- | --- | --- | --- | --- | --- |
| 24 hours | ns | ns | ns | ns | ns | * | *** |
| 48 hours | ** | ns | * | * | ns | ** | **** |
| 72 hours | ns | * | * | **** | **** | *** | **** |
