# Supplementary figures and images for: Bromamine T (BAT) Exerts Stronger Anti-Cancer Properties than Taurine (Tau)
Source: Cancers (Basel). 2021 Jan 7;13(2):182. doi: 10.3390/cancers13020182 (PMC7825693; doi:10.3390/cancers13020182)

**Figure S3:**Whole gel figures of apoptotic cell death in RKO and MDA-MB-468 cancer cells.

(A)


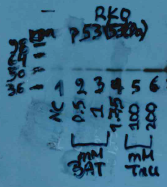


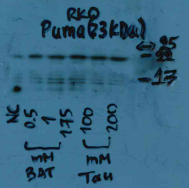


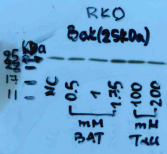


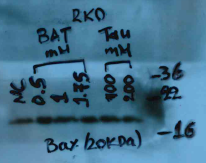


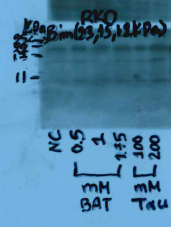


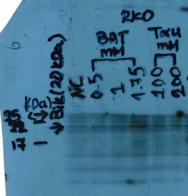


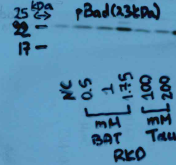


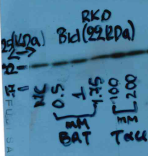


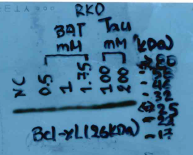


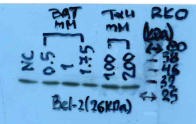


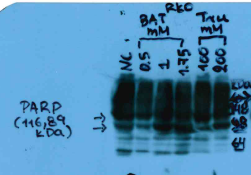


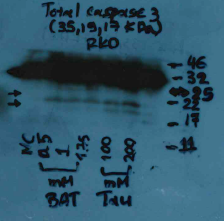


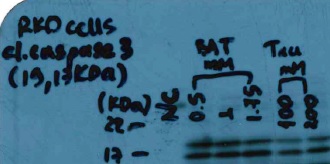


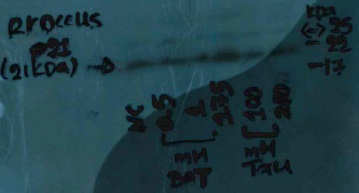


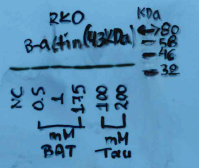


(B)


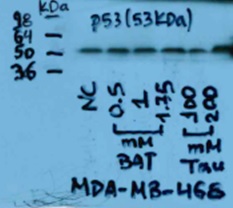


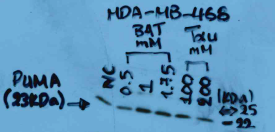


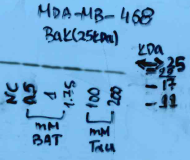


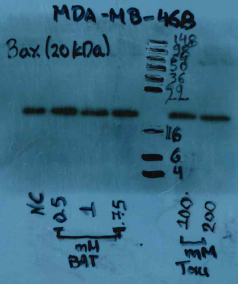


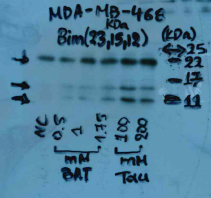


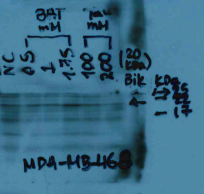


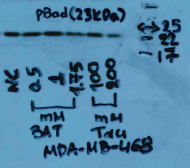


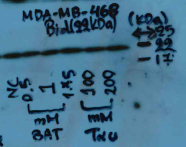


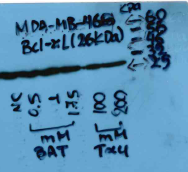


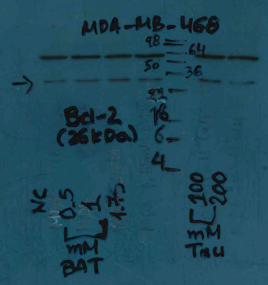


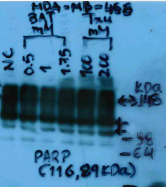


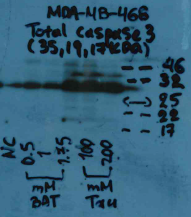


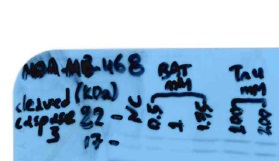


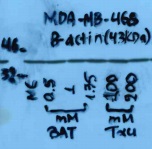

Supplement: Supplementary file 1 [file cancers-13-00182-s001.zip › Figure S3.docx]

**Figure S4.** Whole gel figures of MAPK signaling pathway, autophagy and DDR in RKO cells.

(A)


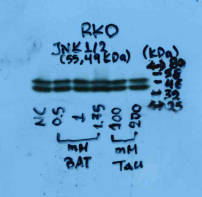


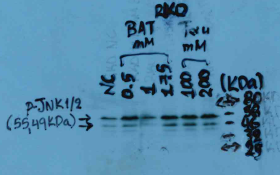


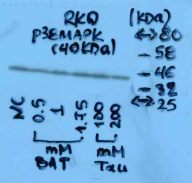


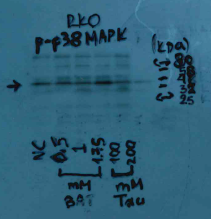


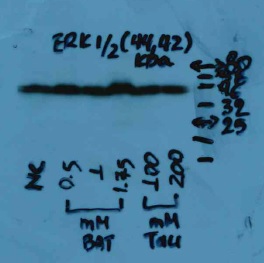


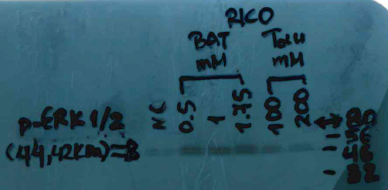


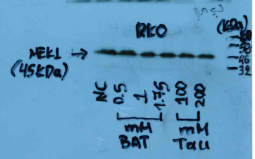


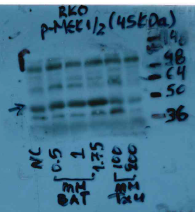


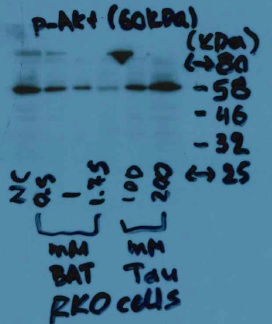


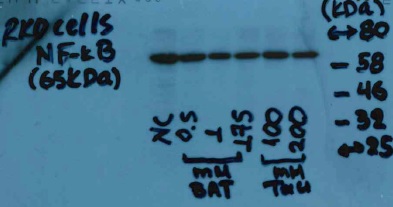


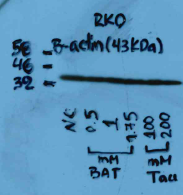


(B)


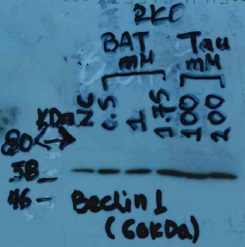


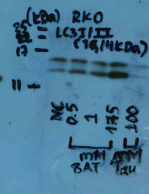


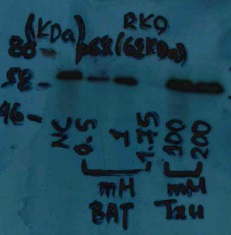


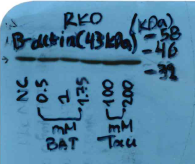


(C)


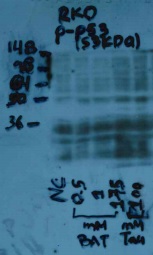


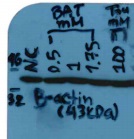

Supplement: Supplementary file 1 [file cancers-13-00182-s001.zip › Figure S4.docx]
